# Cysteine redoxome landscape in the liver of male mice fed a high-fat high-sucrose diet

**DOI:** 10.1016/j.jbc.2025.110730

**Published:** 2025-09-16

**Authors:** Cynthia M. Galicia-Medina, Hein Ko Oo, Takumi Nishiuchi, Ryota Tanida, Tuerdiguli Abuduyimiti, Hisanori Goto, Yujiro Nakano, Yumie Takeshita, Kiyo-aki Ishii, Takashi Toyama, Yoshiro Saito, Hiroaki Takayama, Toshinari Takamura

**Affiliations:** 1 Department of Endocrinology and Metabolism, Kanazawa University Graduate School of Medical Sciences, Kanazawa, Japan; 2 Division of Natural System, Kanazawa University Graduate School of Natural Science and Technology, Kanazawa, Japan; 3 Department of Bone and Joint Disease, National Center for Geriatrics and Gerontology, Obu, Japan; 4 Laboratory of Molecular Biology and Metabolism, Graduate School of Pharmaceutical Sciences, Tohoku University, Sendai, Japan; 5 Life Sciences Division, Engineering and Technology Department, Kanazawa University Graduate School of Medical Sciences, Kanazawa, Japan

**Keywords:** proteomics, liver metabolism, posttranslational modification (PTM), oxidation-reduction (redox), protein motif

## Abstract

Reversible cysteine posttranslational modifications serve as a “switch” for protein structure-function dynamics. Herein, we applied a comprehensive strategy to map the cysteine redoxome by pinpointing over 5000 oxidized and reduced cysteine residues in the liver of male mice fed either a normal chow diet or a high-fat/high-sucrose diet (HFHSD). The global and subcellular distribution of oxidized and reduced cysteine residues remained stable across both diet groups, indicating that HFHSD does not induce widespread shifts in cysteine redox equilibrium. Proteomic analyses revealed that HFHSD upregulates proteins involved in genomic stability, lipid detoxification, and energy regulation, while downregulating those linked to detoxification and metabolic flexibility. Notably, 169 cysteine residues exhibited dynamic redox changes in response to HFHSD, mapping to 35 Kyoto Encyclopedia of Genes and Genomes pathways central to redox balance and energy homeostasis. Motif and structural analyses demonstrated that the reactivity of cysteine residues sensitive to redox stress is dictated by distinct electrostatic microenvironments and subcellular localization. Cysteine residues sensitive to HFHSD-induced oxidation were enriched in mitochondria and cytosol, and cysteine residues sensitive to HFHSD-induced reduction in extracellular regions. Furthermore, cysteine residues sensitive to HFHSD-induced reduction mainly participate in disulfide bond formation and are exposed to the surface of the protein, suggesting roles as molecular switches in protein function. The current cysteine redoxome strategy broadens the disease-associated proteome landscape and provides potential therapeutic target cysteine residues critical for regulating protein functions and interactions relevant to pathophysiology.

Oxidative stress plays a critical role in the pathogenesis of type 2 diabetes and its complications. Oxidative stress derived from dysregulated glucose and lipid metabolisms (1, 2, 3) perturbates endoplasmic reticulum and mitochondrial homeostasis (4, 5), leading to pancreatic β-cell dysfunction with impairment of insulin secretion (6), and hepatic insulin resistance (7). Reactive oxygen species (ROS) play a vital role in oxidative stress through protein glycation (8), lipid peroxidation (9), DNA or RNA damage (10, 11), and protein posttranslational modification (PTM) (12). Elevated ROS exert oxidative stress through cysteine (Cys) redox modifications of proteins (13). For instance, ROS oxidize Cys^32^ and Cys^35^ in the thioredoxin (TRX1) protein and, consequently, remove it from preexisting TRX1–ASK1 complexes, activate ASK1–JNK signaling, and impair insulin signal transduction (13, 14).

On the other hand, physiological levels of ROS are essential for fine-tuning signal transduction and cellular homeostasis (15). Specifically, a transient ROS burst shuts down the activity of protein tyrosine phosphatase 1B by oxidizing the active site Cys^215^ (16), thereby enhancing tyrosine phosphorylation and insulin signaling (17). The intracellular ROS level is balanced between generating (*e.g.* NADPH oxidase 4) (18) and antioxidant systems (*e.g.* SOD, CAT, and selenoproteins family) (19, 20). Selenoprotein P is a 55 kDa secretory protein mainly from the liver and functions as an antioxidant protein through its intrinsic TRX1 domain and by distributing selenium to intracellular selenoproteins, such as glutathione peroxidases (13). Selenoprotein P was rediscovered as a diabetes-associated hepatokine that causes reductive stress by eliminating physiological ROS, leading to insulin resistance in the liver (21), vascular endothelial growth factor resistance in the vessels (22), exercise resistance in the skeletal muscle (23), and thermogenesis resistance in the brown adipose tissue (24). Therefore, to fully understand the molecular mechanisms underlying diabetic pathologies, it is essential to consider not only the targets of oxidative stress but also those of reductive stress.

Cysteine residues of proteins can redox cycle between oxidized and reduced states, known as Cys-PTMs, and impart structural flexibility in the protein due to its sulfur atom. It can be switched between the reduced (thiol) and oxidized forms in response to redox fluctuations (25). The reversible oxidation products of cysteine thiol oxidation includes the formation of disulfide bonds (S-S) (26), S-sulfenylation (S-OH) (27), S-nitrosylation (S-NO) (28), and S-glutathionylation (S-SG) (29). This characteristic is influenced by intramolecular electrostatic forces from neighboring positive- and negative-charged amino acids (30). Indeed, ROS modulate redox signaling, protein structure, and function through the reversible oxidation of cysteine residues (30, 31, 32). Therefore, the reduced and oxidized states of a cysteine residue may function as binary “switches” for regulating protein structures and functions. For example, in brown adipose tissue, the reversible oxidation of Cys^254^ of the uncoupling protein 1 is essential for thermogenesis, which is canceled by selenoprotein P in diabetic condition (24).

The role of reversibly oxidized cysteine residues has been widely explored in the mouse liver in aging (30), high-fat diet (HFD)/fasting (33), and ethanol consumption (34). Nevertheless, research has not covered the impact of reduced cysteine residues on pathology. Specifically, a high-fat/high-sucrose diet (HFHSD) induces obesity, diabetes, insulin resistance, and metabolic dysfunction–associated steatotic liver disease (21, 35), by elevating the levels of intracellular ROS, increasing the oxidation of lipids and proteins, and altering the expression of antioxidant enzymes (36, 37). Simultaneously, HFHSD upregulates the expression of selenoprotein P in the liver, which may lead to protection from oxidative stress and a shift in cellular redox poise toward a more reduced state (21). Therefore, the HFHSD may be a suitable model to observe changes in both protein oxidation and reduction, at least in the liver. Hence, a complete *in vivo* cysteine redoxome profiling, including the identification, evaluation, contribution, and characterization of both reversibly oxidized and reduced cysteine residues, is necessary for a deeper understanding of metabolic homeostasis in the liver. In the present study, we have applied an accessible strategy to evaluate the cysteine redox landscape, taking advantage of detecting both reversibly oxidized and reduced cysteine residues in the liver of male mice fed either a normal chow diet (NCD) or HFHSD. We discovered that subcellular localization, protein structural localization, and electrostatic interactions dictate cysteine residue reactivity. These physicochemical properties influence how redox stress affects specific cellular processes and metabolic pathways in the liver. Understanding the role of cysteine reduction, in addition to cysteine oxidation, could pave the way for manipulating the redox status of diabetes-associated target cysteine residues involved in protein structures and functions.

## Results

### Profiling the liver cysteine redoxome and proteome of mice fed an NCD or HFHSD

**Figure 1:**
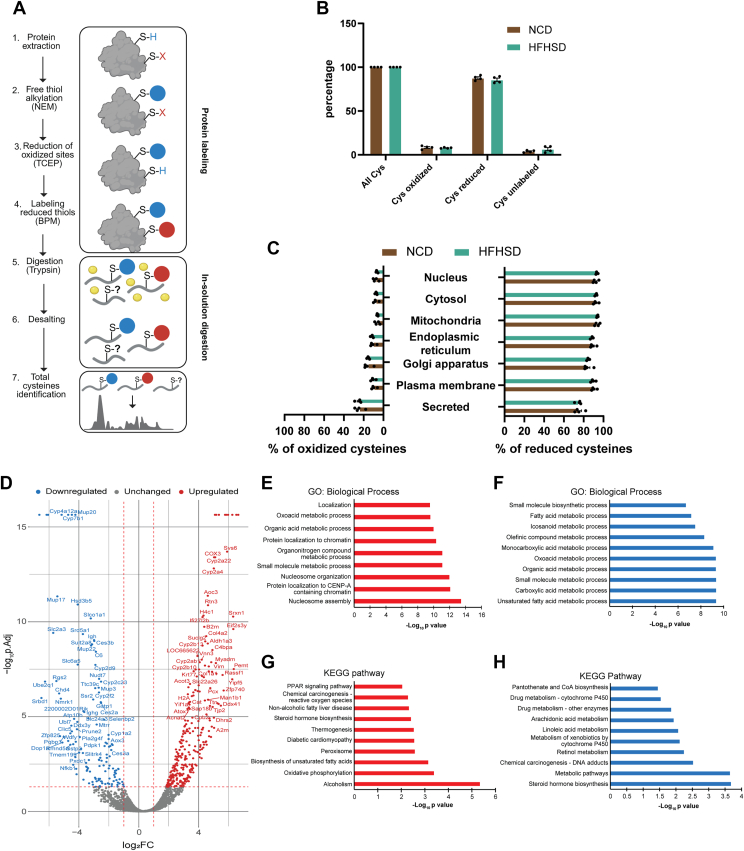
**Cysteine redoxome profile in the liver of mice fed an NCD or HFHSD.***A*, differential alkylation-based bioswitch labeling workflow. (1) Protein was extracted from the liver tissues, (2) native thiol groups (represented as S-H) from cysteines were alkylated using N-ethylmaleimide (NEM, represented in a *blue circle*), (3) the reversibly oxidized cysteine residues (represented as S-X) were reduced with tris(2-carboxyethyl) phosphine (TCEP), (4) the nascent thiol groups (represented as S-H) were labeled using biotin-peac5-maleimide (BPM; represented in a *red circle*), (5) proteins were trypsin digested to produce labeled and unlabeled peptides (represented as S-?), (6) obtained peptides were desalted *via* high pH reversed-phase peptide fractionation to remove impurities, and (7) peptides were prepared for LC-MS analysis to identify total cysteine residues in the sample. *B*, percentage (%) of all reduced or oxidized cysteine residues in the liver in four replicates. *C*, subcellular distribution of oxidized and reduced cysteine residues in the liver in four replicates. *D*, volcano plot depicting changes in protein abundance between NCD *versus* HFHSD groups. The *horizontal red line* indicates a *p* value of 0.05, while the *vertical red line* marks a fold change of 1.0, indicating significantly increased or decreased based on the volcano plot. *Blue dots* represent proteins downregulated in HFHSD, *red dots* represent proteins upregulated in HFHSD, and *gray dots* indicate protein with unchanged expression in HFHSD. *E*, relevant enriched GO: BPs of proteins significantly increased in HFHSD, based on the volcano plot (D). *F*, relevant enriched GO: BPs of proteins significantly decreased in HFHSD, based on the volcano plot (D). *G*, relevant enriched KEGG pathways of proteins significantly increased in HFHSD, based on the volcano plot (D). *H*, relevant enriched KEGG pathways of proteins significantly decreased in HFHSD based on the volcano plot (D). The protein abundances and complete list of enriched GO:BPs, and KEGG pathways can be found in Tables S1–S6. In the GO analysis graphs, *blue bars* indicate proteins significantly decreased in HFHSD, while the *red bars* indicate proteins significantly increased in HFHSD. In (*B*) and (*C*), data are represented as mean ± SD. Statistical significance is calculated by two-tailed unpaired student’s *t* test. There is no significant difference between NCD and HFHSD groups in (*B*) and (*C*). BP, biological process; GO, gene ontology; HDHSD, high-fat/high-sucrose diet; KEGG, Kyoto Encyclopedia of Genes and Genomes; NCD, normal chow diet.

We first investigated the cysteine redoxome profile in the liver of male mice fed a NCD or HFHSD and consequently performed a differential alkylation-based proteomics technique without denaturing agent (Fig. 1*A*). We analyzed the livers of C57BL/6 mice fed NCD or HFHSD for 16 weeks, composed of 4 mice each group (Fig. S1). We categorized the cysteine residues as follows: reduced cysteines were identified based on reactivity with N-ethylmaleimide (NEM); reversibly oxidized cysteines were identified by reactivity with biotin-peac5-maleimide (BPM) after reduction; and unlabeled cysteine. Only 3.9% and 6.5% of cysteine residues in the NCD and HFHSD groups, respectively, were unlabeled (Fig. 1*B*), demonstrating the efficiency of the differential alkylation method for labeling these sites; in both cases, >90% of the cysteine residues were labeled according to their redox status. The percentage of oxidized and reduced cysteine residues was not different in the HFHSD compared to the NCD group (Fig. 1*B*).

To understand the subcellular distribution of proteins with identified cysteine residues, we classified proteins based on their oxidized and reduced cysteine states from both diet groups *via* their subcellular compartments using subcellular localization databases (Fig. 1*C*). The HFHSD group and NCD group shared a similar profile in the percentage of oxidized and reduced cysteine residues across all subcellular compartments. For instance, the percentage of oxidized cysteines was not altered in mitochondria with 5.9% in the NCD group and 6.2% in the HFHSD group. Similar results with the percentage of oxidized cysteines in the endoplasmic reticulum 10.9% in the NCD group and 10.7% in the HFHSD group (Fig. 1*C*).

Next, we analyzed protein abundance differences between the NCD and HFHSD groups using a volcano plot (Fig. 1*D*). Significantly altered proteins were subjected to gene ontology (GO) enrichment analysis and Kyoto Encyclopedia of Genes and Genomes (KEGG) pathway mapping (Fig. 1, *E* and *F*). We found 475 proteins upregulated and 208 proteins downregulated in the HFHSD group (Tables S1 and S2). The proteins upregulated in the HFHSD group showed enrichment in biological processes (BPs) such as nucleosome assembly, nucleosome organization, oxoacid metabolic process, and protein localization to chromatin (Fig. 1*E* and Table S3). On the other hand, the downregulated proteins in the HFHSD group were linked to lipid and organic acid metabolism pathways, including unsaturated fatty acid metabolic process, carboxylic acid metabolic process, organic acid metabolic process, and monocarboxylic acid metabolic process (Fig. 1*F* and Table S4). The KEGG enrichment analysis of upregulated proteins in the HFHSD group revealed associations with alcoholism, oxidative phosphorylation, thermogenesis, nonalcoholic fatty liver disease, and peroxisome proliferator-activated receptors (PPAR) signaling pathway (Fig. 1*G* and Table S5). Downregulated proteins in the HFHSD group were enriched in steroid hormone biosynthesis, retinol metabolism, linoleic acid metabolism, arachidonic acid metabolism, and pantothenate and CoA biosynthesis (Fig. 1*H* and Table S6).

### Classification of HFHSD-sensitive cysteine residues and their functional roles in liver metabolic pathways and redox regulation

**Figure 2:**
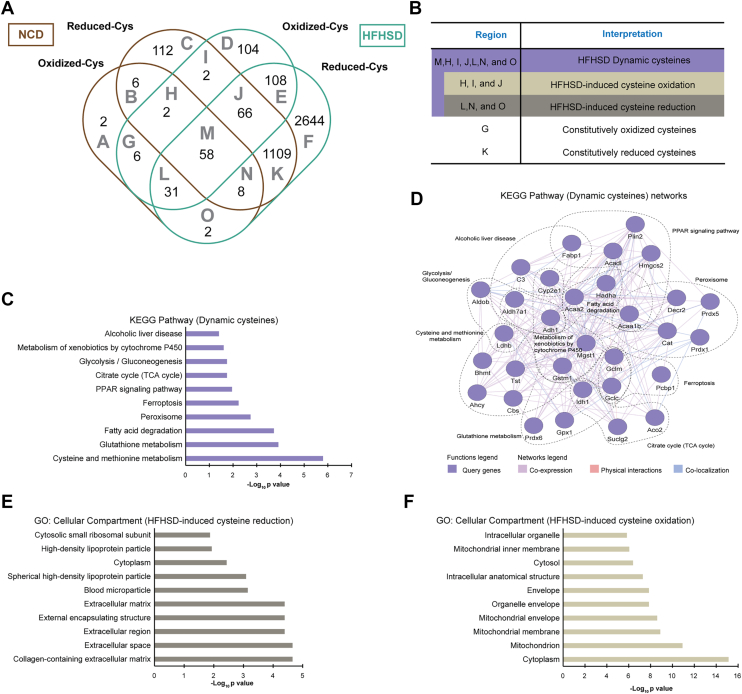
**Identifying GO terms and KEGG pathways in HFHSD-sensitive cysteine residues.***A*, Venn diagram showing the overlap and exclusivity of reproducible oxidized and reduced cysteine residues validated in quadruplicate experiments in each diet model as shown in Fig. S1. *B*, table describing the interested regions of the Venn diagram and their interpretations under an HFHSD. *C*, ten relevant enriched KEGG pathways observed in HFHSD dynamic cysteine residues in NCD and HFHSD groups. A comprehensive list of enriched terms is available in Table S8. *D*, network predicting protein interactions containing HFHSD dynamic cysteine residues using GeneMANIA. The legend within the network illustrates the type of connections between genes/proteins. Enrichment analyses based on CCs (E-F) found in the reactive cysteine residues sensitive to HFHSD-induced reduction and oxidation. The complete lists of enriched terms are presented in Tables S9 and S10. GO, gene ontology; HDHSD, high-fat/high-sucrose diet; KEGG, Kyoto Encyclopedia of Genes and Genomes; NCD, normal chow diet; CC, cellular compartment.

Next, only the cysteine residues detected consistently in at least 2 out of 4 independent replicates within the same diet group and under the same redox category (oxidized or reduced) were considered to eliminate possible “false-positive” hits (Fig. S1). Our studies identified 7 proteins previously established to exhibit cysteine thiol oxidation, including glutathione peroxidase 1, fatty acid–binding protein 1 (FABP1), liver, and mitochondrial carnitine/acylcarnitine translocase (Fig. S2). In addition, we assessed the biotinylation status of FABP1 and serum paraoxonase/arylesterase 1 (PON1) as representative proteins of HFHSD-induced cysteine oxidation and reduction, respectively. Both showed significant changes in their respective modifications in the HFHSD group (Figs. S3 and S4). The consistently detected cysteine residue sites included 115 and 377 oxidized and 1363 and 4026 reduced cysteine residue sites in the NCD and HFHSD groups, respectively, illustrated in a Venn diagram (Fig. 2*A* and Table S7). We classified cysteine residues into five distinct categories based on their reactivity to HFHSD (Fig. 2*B*). These categories include 1) HFHSD dynamic cysteines, which represent cysteine residues that change their redox status due to HFHSD; 2) HFHSD-induced cysteine oxidation, which represent cysteine residues that become more oxidized due to HFHSD; 3) HFSHD-induced cysteine reduction, which are cysteine residues that become more reduced due to HFHSD; 4) constitutively oxidized; and 5) constitutively reduced cysteines in both diet groups.

We identified 169 HFHSD dynamic cysteine residues that exhibited changes in redox state in response to the diet, accounting for approximately 4% of consistently identified cysteines (Fig. 2*A*). We addressed the significance of these HFHSD dynamic cysteine residues in metabolism by performing KEGG enrichment analysis. KEGG enrichment analysis unveiled 35 metabolic pathways relevant to cysteine and methionine metabolism, glutathione metabolism, fatty acid degradation, PPAR signaling pathway, tricarboxylic acid (TCA) cycle, gluconeogenesis, and alcoholic liver disease (Fig. 2*C* and Table S8). The protein association network containing HFHSD dynamic (Fig. 2*D*) revealed robust interconnectivity among key metabolic regulators, including glutathione s-transferase, glutamate-cysteine ligase (GCL), alcohol dehydrogenase 1, and ACAA2 (3-ketoacyl-CoA thiolase, mitochondrial). These proteins exhibited strong functional associations *via* coexpression, physical interactions, and colocalization (Fig. 2*D*), in addition to their enriched KEGG pathways, suggesting that HFHSD dynamic cysteine residues act as coordinated redox sensors, modulating cellular responses to diet-induced metabolic stress.

Further analysis was performed to investigate the roles of cysteines, associated with HFHSD-induced oxidation or reduction, in cellular compartment (CC), BP, and molecular function (MF). In the CC category, we discovered 34 CCs associated with the cysteines sensitive to HFHSD-induced reduction, among them the extracellular region and cytoplasm prevailed (Fig. 2*E* and Table S9). Conversely, 50 CCs found in the reactive cysteines sensitive to HFHSD-induced oxidation, these reactive cysteines-containing proteins are located in the cytoplasm, mitochondrion, and mitochondrial organelles (Fig. 2*F* and Table S10). We identified in the cysteines sensitive to HFHSD-induced reduction 180 BPs associated with toxic substances, organonitrogen compound metabolic process, phosphorus metabolic process, and regulation of lipid localization (Fig. S5*A* and Table S11). In contrast, cysteines sensitive to HFHSD-induced oxidation were associated with 525 BPs, including the response to energy production and detoxification in the liver, such as oxoacid metabolic process, organic acid metabolic process, and carboxylic acid metabolic process. Additionally, BPs critical for mitochondrial function, such as small molecule metabolic and catabolic processes, were significantly enriched (Fig. S5*B* and Table S12). Analysis of MFs identified 122 and 144 enriched in cysteines linked to HFHSD-induced reduction or oxidation, respectively. For the cysteines associated linked response to HFHSD-induced reduction, MFs such as guanyl nucleotide binding, GTP binding, peptidase inhibitor activity, endopeptidase inhibitor activity, TRX1-dependent peroxiredoxin activity, and enzyme inhibitor activity involved in vesicle transport, protein aggregation, endoplasmic reticulum stress, and ROS signaling balance (Fig. S5*C* and Table S13). NAD binding, heterocyclic compound binding, oxidoreductase activity, ligase activity forming carbon-nitrogen bonds, and catalytic activity are involved in the depletion of critical cofactors (*e.g.*, NAD^+^) and damage of proteins in the cysteines associated to HFHSD-induced oxidation (Fig. S5*D* and Table S14). These findings highlight distinct roles of cysteines under HFHSD-induced reduction and oxidation conditions, reflecting their involvement in maintaining redox balance and regulating liver metabolism.

### Distinct motifs for HFHSD-sensitive cysteine residues

To determine whether specific amino acid motifs are associated with HFHSD dynamic cysteines, HFHSD-induced cysteine reduction, or HFHSD-induced cysteine oxidation, we aligned peptide sequences within ±7 amino acids surrounding the cysteine residues and performed peptide sequence motif analyses. Consistent with prior reports linking cysteine redox sensitivity to proximal charged residues (30, 32), HFHSD dynamic cysteine residues exhibited enrichment of positively charged lysine (Lys; K) at the −1 position (Fig. S6*A*). Position weight matrix (PWM) heat maps further revealed distinct amino acid property patterns for HFHSD dynamic cysteines (Fig. S6*B*). Negatively charged aspartic acid (Asp; D) was prevalent present at positions −5, −1, +2, and +6, while glutamic acid (Glu; E) dominated positions −5, −4, −3, +1, +2, +3, +4, +5, and +7. On the other hand, polar uncharged residues cysteine (Cys; C) and serine (Ser; S) were underrepresented across all the ± 7.

Next, we analyzed peptide motifs associated with cysteines sensitive HFHSD-induced reduction or oxidation to gain deeper insights. The cysteines targeted by HFHSD-induced reduction lacked significant motifs (Fig. S6*C*) but displayed unique patterns (Fig. S6*D*), including polar residues such as polar amino acids threonine (Thr; T), proline (Pro; P) at the −7, −3, −2, +1, +,4, +5 positions. In addition to nonpolar amino acids valine (Val; V), and methionine (Met; M) at the positions −6, −5, −3, −2, and +6. The underrepresented amino acids also displayed positively charged amino acid arginine (Arg; R) at the positions −7, −4, ±1, and nonpolar amino acids C, S, and Q at the −6, ±3, ±5 positions (Fig. S6, *C* and *D*). Conversely, constitutively reduced cysteine residues showed an enrichment of negatively charged amino acids E and D at the ± 6 positions and did not exhibit enrichment of nonpolar or polar amino acids (Fig. S6, *E* and *F*) compared to HFHSD-induced cysteine reduction.

**Figure 3:**
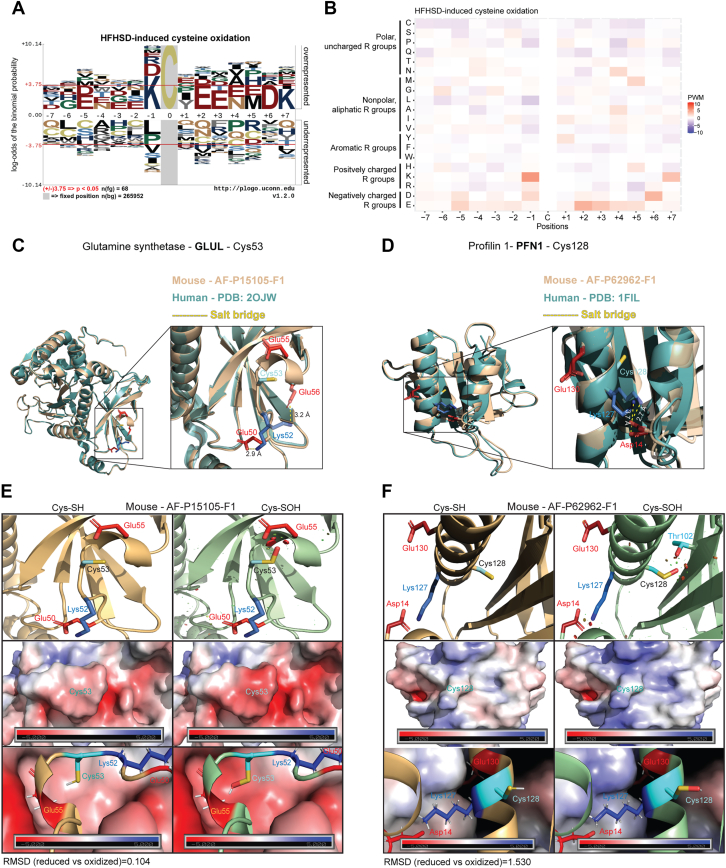
**Motif analysis of cysteines sensitive to HFHSD-induced oxidation.***A*, sequence motif analysis of cysteines sensitive to HFHSD-induced oxidation, examining proximal positions (±7 positions) relative to the cysteine sites. *Horizontal red lines* indicate significance at a *p* value of 0.05. *B*, heat map illustrating amino acid sequences surrounding HFHSD dynamic cysteine residues (*A*). The heat map displays position weight matrix (PWM) values, reflecting amino acid frequencies proximal to the cysteine and categorized by amino acid properties. *C*-*F*, protein structures of HFHSD-induced cysteine oxidation containing the KCxE motif. *C*, overlayed structure of GLUL: mouse (AlphaFold), human (PDB: 2OJW). The inset shows KCxE motif residues participation in salt bridge formation between Lys, Glu, and Asp amino acids. Salt bridges are marked with *dashed yellow lines* and distance is shown in angstrom (Å). *D*, overlayed structure of PFN1: mouse (AlphaFold), human (PDB: 1FIL). The inset shows “KCxE” motif residues participation in salt bridge formation between Lys and Asp amino acids. Salt bridges are marked with *dashed yellow lines*, and distance is shown in angstrom (Å). *E*, electrostatic surface potential of GLUL in mouse (AlphaFold). *Left:* Structure with reduced Cys^53^ (Cys-SH). *Right:* Structures with oxidized Cys^53^ (Cys-SOH) modeled with PyTMs plugin in PyMOL. The PDB2PQR webtool was used to visualize the electrostatic surface, for oxidized Cys^53^ (Cys-SOH) a customized AMBER-forcefield parameters was used (Fig. S9). Steric van der Waals (vdW) hindrance (vdW clashes) is shown in *red*. *F*, electrostatic surface potential of PFN1 in mouse (AlphaFold). *Left:* Structure with reduced Cys^128^ (Cys-SH). *Right:* Structures with oxidized Cys^128^ (Cys-SOH) modeled with PyTMs plugin in PyMOL. The PDB2PQR webtool was used to visualize the electrostatic surface, for oxidized Cys^128^ (Cys-SOH) a customized AMBER-forcefield parameters was used (Fig. S9). Steric vdW hindrance (vdW clashes) is shown in *red*. Cys residues are shown in *cyan*, Lys residues are shown in *blue*, Glu and Asp residues are shown in *red*, and Thr residues are shown in *cyan*. Side chains of amino acids are presented in *sticks*. RMSD, root mean square deviation of atomic positions. HDHSD, high-fat/high-sucrose diet; PFN1, profilin 1.

Cysteines related to HFHSD-induced oxidation showed an enrichment of basic amino acid K at the −1 position and acidic amino acid E at the +2 position (Fig. 3*A*). In the KCxE motif, charged neighboring residues might influence cysteine’s reactivity through charge modulation or hydrogen bonding (30, 32). We observed a higher prevalence of acidic amino acids E at −5, +3 positions; D at −1, +6 positions, and basic amino acids K at the +7 position (Fig. 3*B*). Polar amino acid glutamine (Gln; Q) and nonpolar amino acid leucine (Leu; L) were underrepresented −6, ±1, +2, and ±7 positions. Differently, constitutively oxidized residues did not exhibit enrichment of basic or acidic amino acids in comparison to HFHSD-induced cysteine oxidation (Fig. S6, *G* and *H*).

Next, to understand the KCxE motif in the HFHSD-induced cysteine reduction group, we performed *in silico* molecular analysis of the steric van der Waals (vdW) interactions and electrostatic surface potential changes between the reduced and oxidized cysteine residues in two proteins containing the identified consensus motif, glutamine synthetase (GLUL) and profilin 1 (PFN1). GLUL in the liver plays a role as one of the enzymes responsible for the removal of ammonia (38). PFN1 is an actin-binding protein crucial for actin polymerization and maintains mitochondrial integrity and function (39). The Cys^53^ in GLUL and Cys^128^ PFN1 are conserved in mice and humans, and form salt bridges with nearby negatively charged amino acids, Glu and Asp (Fig. 3, *C* and *D*). We constructed the GLUL and PFN1 structures with sulfenylated (-SOH) Cys^53^ and Cys^128^ residues, respectively, to generate the oxidized forms (Cys-SOH). The reduced forms (Cys-SH) of GLUL and PFN1 at these positions served as controls for comparing electrostatic changes induced by Cys-SOH. The oxidized Cys^53^ in GLUL from both mouse and human showed predicted steric clashes (vdW clashes) with Glu^55^ (Figs. 3*E* and S7*A*). Similarly, oxidized Cys^128^ in PFN1 exhibited vdW clashes with Thr^102^ in both species and additionally between Lys^127^ and Asp^14^ in mouse (Figs. 3*F* and S7*B*). Electrostatic surface analysis revealed differences in the electrostatic potential between the oxidized and reduced forms of Cys^53^ in GLUL (Figs. 3*E* and S7*A*) and Cys^128^ in PFN1 (Figs. 3*F* and S7*B*), suggesting a likely conformational change following oxidative modification of these cysteine residues. Together, these findings suggest that cysteines sensitive to HFHSD-induced oxidation are flanked by charged amino acid motifs, whereas reactive cysteines sensitive to HFHSD-induced reduction are flanked by nonpolar/polar residues. The significance of these characteristic features is unknown but may reflect distinct redox reactivity mechanisms.

### Physicochemical properties of reactive cysteine residues in HFHSD

**Figure 4:**
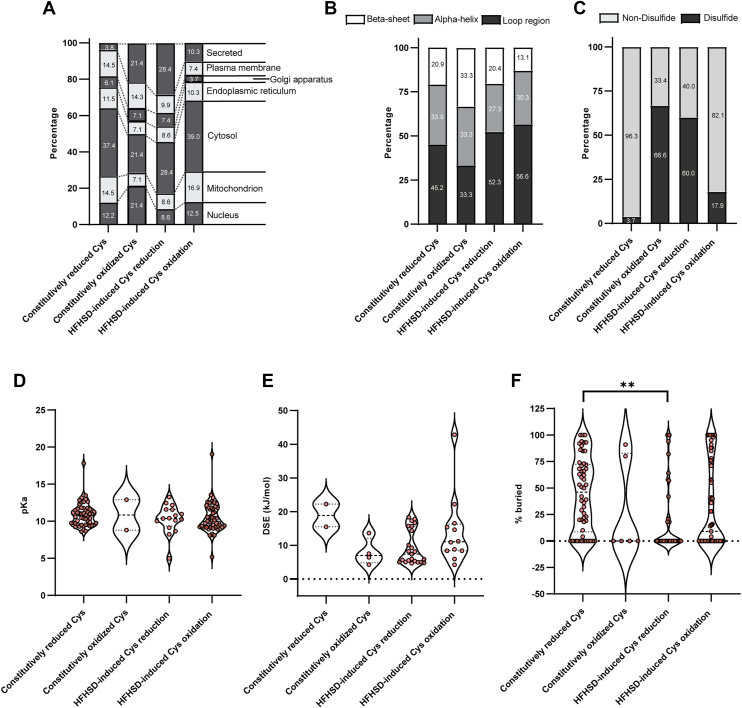
**Subcellular localization and physicochemical properties of reactive cysteine residues in HFHSD.** Due to the abundance of constitutively reduced cysteines (1109 sites; Fig. 2, *A* and *B*) in both diets, we used the KC motif (identified in HFHSD dynamic cysteines; Fig. 3*B*) to filter these residues. The resulting KC-containing constitutively reduced cysteines served as a control group, together with constitutively oxidized cysteines, for comparison to reactive cysteine residues responsive to reductive/oxidative stress. *A*, subcellular localization of proteins containing (i) constitutively reduced, (ii) constitutively oxidized cysteine residues in NCD and HFHSD groups, (iii) HFHSD-induced cysteine reduction, (iv) HFHSD-induced cysteine oxidation. *B*, structural location within proteins of (i) constitutively reduced, (ii) constitutively oxidized cysteine residues in NCD and HFHSD groups, (iii) HFHSD-induced cysteine reduction, (iv) HFHSD-induced cysteine oxidation. *C*, the nature of constitutively reduced and oxidized cysteine residues in NCD and HFHSD groups, and cysteine residues sensitive to HFHSD-induced reduction and oxidation. Disulfide, cysteine residues involved in intramolecular disulfide bond formation; non-disulfide, cysteine residues not involved in intramolecular disulfide bond formation. *D*, violin plot of the estimated pKa values of constitutively reduced and oxidized cysteine residues in NCD and HFHSD groups, and cysteine residues sensitive to HFHSD-induced reduction and oxidation. *E*, violin plot of the estimated dihedral energy strain (DSE) values of disulfide bonds in constitutively reduced and oxidized cysteine residues in NCD and HFHSD groups, cysteine residues sensitive to HFHSD-induced reduction and oxidation. *F*, violin plot of the estimated buried percentage of constitutively reduced and oxidized cysteine residues in NCD and HFHSD groups, and cysteine residues sensitive to HFHSD-induced reduction and oxidation. For the violin plots of Figure 4, *D*–*F*, statistical differences among the four cysteine residue categories were assessed using one-way ANOVA tests followed by Tukey’s *post hoc* test. ∗*p* ≤ 0.05. HDHSD, high-fat/high-sucrose diet; NCD, normal chow diet.

Next, further analysis of cysteine residues sensitive to HFHSD-induced oxidation or reduction was performed to elucidate their subcellular localization, intramolecular localization, and physicochemical properties compared to the constitutively reduced and oxidized cysteine residues in an HFHSD. Constitutively redox-stable cysteine residues were localized to the cytosol (37.4% reduced, 21.4% oxidized) (Fig. 4*A*). Constitutively oxidized residues also showed a prevalent presence in the nucleus (21.4%) and extracellular compartment (21.4%). In contrast, HFHSD-induced cysteine reduction group was localized primarily to the cytosol (28.4%) and extracellular compartment (28.4%), while HFHSD-induced cysteine oxidation group was most abundant in the cytosol (39.0%) and mitochondria (16.9%). The protein structural localization of constitutively reduced cysteine residues was predominantly located at the loop region (45.2%), while constitutively oxidized cysteine residues were uniformly distributed across the β-sheets (33.3%), α-helices (33.3%), and loop regions (33.3%) (Fig. 4*B*). Cysteine residues associated with HFHSD-induced reduction or oxidation were similarly localized to loop regions (52.3% and 56.6%, respectively), suggesting that structural flexibility in these regions may facilitate redox-sensitive interactions.

Among cysteine residues sensitive to HFHSD-induced reduction, 60% were found to participate in intramolecular disulfide bond formation (Fig. 4*C*). Notable examples include the disulfide bonds between Cys^42^ and Cys^353^ in PON1 and between Cys^211^ and Cys^179^ of cathepsin B (CTSB), which are also conserved in humans (Fig. S8, *A* and *D*). Electrostatic surface analysis revealed no significant differences in the electrostatic potential at the reduced forms of Cys^42^ in PON1 and Cys ^211^ in CTSB compared to their respective disulfide bond states (Cys^42^-^353^ in PON1, Fig. S8, *B*–*C*, and Cys^211^-Cys-^179^ in CTSB, Fig. S8, *E*–*F*). In contrast, only 3.7% of constitutively reduced cysteine residues and 17.9% of cysteine residues sensitive to HFHSD-induced oxidation contributed to disulfide bond formation. Cysteine residues sensitive to HFHSD-induced oxidation were predominantly noninvolved in disulfide bond formation (82.1% nonparticipating). Conversely, only 33.4% of constitutively oxidized cysteine residues did not participate in disulfide bonds. Similarly, among cysteine residues targeted by HFHSD-induced reduction, 40% were classified as non-disulfide bond–forming residues. The estimated pKa values showed no significant differences between constitutive or HFHSD-induced cysteine categories, with values ranging from 8 to 13 across all categories (Fig. 4*D*). Similarly, dihedral strain energy, which quantifies the torsional strain associated with the five dihedral angles along the sulfur–sulfur bonds in cysteine residues, exhibited no statistically significant variation among categories, averaging 10 to 20 kJ/mol (Fig. 4*E*). However, the cysteine residue accessibility differed significantly in the reduced cysteine residue categories. Cysteine residues sensitive to HFHSD-induced reduction were mainly surface-exposed (0% buried), whereas constitutively reduced cysteine residues were predominantly embedded within the protein core (Fig. 4*F*). Our analyses revealed distinct subcellular and structural localizations, as well as varied participation in disulfide bond formation, among cysteine residues to HFHSD-induced oxidation or reduction, highlighting their reactivity in redox conditions and properties in protein function.

## Discussion

The present study has applied a straightforward strategy for labeling cysteine residues and reported a cysteine redox landscape encompassing over 5000 oxidized and reduced cysteine residues in the liver of male mice fed NCD and HFHSD. The percentage of reduced and oxidized cysteine residues remained stable between mice fed an NCD and HFHSD. In addition, the conserved patterns of percentage of oxidized and reduced cysteine residues across all subcellular compartments prevailed unchanged in both diet groups. These findings suggest that HFHSD does not affect the global and subcellular shift in the redox equilibrium of cysteine residues.

GO BPs analyses revealed that the upregulated proteins in the HFHSD group are enriched in processes maintaining genomic stability, epigenetic regulation of gene expression, lipid detoxification, energy regulation, and bile acid homeostasis. Moreover, GO BPs analyses disclosed that the downregulated proteins in the HFHSD group are enriched in impairing detoxification, suppressing lipid oxidation, and disrupting metabolic flexibility. The upregulated proteins in the HFHSD group aligned with multiple KEGG pathways, reflecting metabolic dysregulation (including the PPAR signaling pathway and thermogenesis) and disease progression (such as nonalcoholic fatty liver disease and diabetic cardiomyopathy). These KEGG pathways were distinct from those associated with downregulated proteins, which primarily reflected impaired metabolic flexibility (*e.g.*, pantothenate and CoA biosynthesis, retinol metabolism) and reduced detoxification capacity (such as cytochrome P450 and metabolism of xenobiotics by cytochrome P450). The collective dysregulation of these BPs and KEGG pathways contributes to the development and progression of hepatic metabolic disorders.

Our analysis identified 169 HFHSD dynamic cysteine residues that undergo redox state changes in response to dietary stress, representing a coordinated network of redox sensors in metabolic regulation. These dynamic cysteine residues mapped 35 KEGG pathways, including GSH metabolism, PPAR signaling, fatty acid degradation, glycolysis/gluconeogenesis, and the TCA cycle, showing their centrality in bridging redox balance (40) with energy homeostasis (41, 42, 43, 44). The protein interaction network revealed strong functional associations among key metabolic regulators such as GCLC and GCLM, the two subunits of GCL. GCL enzyme catalyzes the rate-limiting step in the synthesis of the cellular antioxidant GSH (45). Additionally, ACAA2 has an important role in fatty acid metabolism by catalyzing the final step of mitochondrial β-oxidation, a process regulated through PPAR signaling (46). The cysteine residues sensitive to HFHSD-induced reduction were localized in the cytoplasm and extracellular regions. Furthermore, the enriched BPs were related to lipid localization and nitrogen metabolism, with MFs including GTP binding and TRX1-dependent peroxiredoxin activity. The target cysteine residues sensitive to HFHSD-induced reduction play vital roles in regulating vesicle transport (47), neutralizing peroxides (48), and preventing protein aggregation (49). Indeed, our results align with the previous findings that reported the TRX1 system protects against reductive stress in *Saccharomyces cerevisiae* (49). Conversely, the cysteine residues sensitive to HFHSD-induced oxidation were localized to the cytoplasm and mitochondria, enriching processes like oxoacid metabolic process, small molecule metabolic process, NAD binding, and oxidoreductase activity. The target cysteine residues sensitive to HFHSD-induced oxidation highlight their role in sustaining energy production (50) and maintaining redox balance (51, 52).

The motif analyses revealed that HFHSD dynamic cysteine residues and cysteine residues sensitive to HFHSD-induced oxidation are governed by distinct electrostatic environments. In contrast, the cysteine residues sensitive to HFHSD-induced reduction exhibited unique nonpolar/polar amino acid patterning. At physiological pH, cysteine thiol(-SH)/thiolate(-S^-^) equilibrium depends on nearby charged residues. The enrichment of K at the −1 position in the HFHSD dynamic cysteine residues and cysteine residues sensitive to HFHSD-induced oxidation stabilizes the thiolate state (30, 32). Aligning with redox sensitivity observed in cold-exposed dynamic cysteine residues in brown adipose tissue (53). This electrostatic effect is further supported by the prevalence of E at the +2 position in cysteine residues sensitive to HFHSD-induced oxidation. This creates a charge microenvironment stabilizing transient oxidative PTMs of cysteine residues, potentially disrupted by K-E salt bridge interference, which is crucial for protein stability and folding (54). Notably, the electrostatic surface analyses revealed changes in the electrostatic surface potential of selected proteins, suggesting that the KCxE motif observed might be a potential target of oxidative stress that could influence protein interactions and activity. On the other hand, cysteine residues sensitive to HFHSD-induced reduction lack charged amino acids (*e.g.*, R), but instead feature V and M at the positions −6 and −5, suggesting hydrophobic clustering or steric constraints that might limit accessibility to oxidants while favoring the reduction. These corroborate findings that oxidized cysteine residues are found much less frequently near hydrophobic amino acids (55).

The compartmentalization and structural dynamics of reactive cysteine residues under HFHSD reveal specialized roles in redox adaptation. Cysteine residues sensitive to HFHSD-induced oxidation are localized predominantly to mitochondria and cytosol. In contrast, cysteine residues sensitive to HFHSD-induced reduction are localized to extracellular regions. Both results confirm the previous findings in the CC category obtained from the GO analyses and suggest determined participation in processes according to their redox-stress stimuli. Cysteine residues sensitive to HFHSD-induced oxidation and reduction are the majority localized in loop regions; this feature supports structural flexibility and interaction with other molecules (56, 57). Moreover, 60% of the cysteine residues sensitive to HFHSD-induced reduction participate in disulfide bond formation and are mostly surface-exposed. The electrostatic surface analysis revealed no significant changes in the electrostatic potential of selected disulfide bonds of secreted proteins. This lack of change might be because disruption of the disulfide bonds does not drastically alter the overall distribution of surface charges or the electrostatic potential detectable at solvent-accessible regions. Nevertheless, previous studies have reported that mutating the Cys^42^-Cys^353^ disulfide bond in PON1 can abolishes the activity and significantly decreases the secretion of the protein (58). These findings suggest that these disulfide bonds might act as switches in protein conformational changes or functions, as reported about the disulfide bond (Cys^155^-Cys^184^) of the glycoprotein CD4 (59). Further studies, such as molecular dynamic simulations and site-directed amino acid mutagenesis, are needed to test this hypothesis in cysteine residues sensitive to HFHSD-induced reduction. The lack of differences in pKa or dihedral strain energy across cysteine residue categories suggests redox sensitivity is governed by microenvironmental factors, such as charged amino acid proximity, rather than intrinsic thermodynamic properties.

Consistent with a previous cysteine proteome study that used an HFD in the liver of mice to identify oxidized cysteine residues (33), we identified 13 cysteine residues among those reactive to HFHSD-induced oxidation conditions (Table S15). Notably, Cys^69^ of FABP1 was identified as a target of oxidative stress under HFD and HFHSD conditions. FABP1 is primarily involved in the uptake, transport, and metabolism of long-chain fatty acids in the liver. It is associated with several liver diseases, including metabolic dysfunction–associated steatotic liver disease (60), and type 2 diabetes (61). Cys^69^ in FABP1 may play a critical role in forming an intermolecular disulfide bond with Cys^81^ in anterior gradient 2, enabling their direct interaction, potentially influencing FABP1 stability and facilitating lipid accumulation (62). On the other hand, we identified 8 cysteine residues sensitive to HFHSD-induced reduction (Table S16), which had previously been identified as oxidized cysteine residues in the previous cysteine proteome study using HFD (33). Remarkably, 4 cysteine residues sensitive to HFHSD-induced oxidation are involved in the formation of intramolecular disulfide bonds. The reduction of these disulfide bonds suggests the presence of redox-active sites within the proteins. However, further functional analyses are required to confirm this hypothesis and to clarify the molecular mechanisms through which these targeted proteins and their reduced cysteine residues contribute to pathological conditions associated with obesity and diabetes. In this study, the proteins containing cysteine residues sensitive to HFHSD-induced oxidation, and also identified in the previous study, showed no change in abundance compared to those in the NCD group (Table S15). This contrasts with the previous study, which reported upregulation of these same proteins in the HFD group compared to the NCD group. Nevertheless, both the cysteine residues sensitive to HFHSD-induced oxidation and the oxidized cysteine residues identified under HFD are involved in similar metabolic pathways, including glycolysis, fatty acid degradation, and the TCA cycle. Furthermore, this study newly identifies 90 cysteine residues that are sensitive to both HFHSD-induced reduction and oxidation, a finding unrecognized in prior HFD research. The discovery of these cysteine residues, which were undetected in earlier work, highlights the importance of mapping oxidized and reduced cysteines within the redoxome landscape to uncover novel targets of liver metabolic stress.

Many studies have used biotin-maleimide to alkylate the oxidized cysteine residues and enriched them for the LC-MS analysis (30, 31, 63). Such an approach in combination with the tandem mass tag can provide the percent oxidation of specific cysteine residues. However, our study analyzed the biotin-labeled and NEM-labeled cysteine residues without streptavidin enrichment before LC-MS analysis. Our methodology can simultaneously profile oxidized and reduced cysteines, rather than measuring percent oxidation. Although the number of cysteine residues that can be detected may greatly decrease without enrichment, the simultaneous detection of both oxidized and reduced cysteines enables us to identify not only the HFHSD-induced cysteine oxidation but also the HFHSD-induced cysteine reduction.

Previous studies (30, 31, 63), including the current one, face a common challenge in accurately quantifying reversibly oxidized cysteine residues, particularly S-persulfidation (S-SH). The alkylating agent NEM can react with the thiol group in S-persulfidated cysteines (S-S-NEM), leading to their misidentification as reduced cysteines (S-NEM) instead of oxidized (32). Future cysteine redoxome profiling in the liver should employ selective labeling techniques to mitigate this issue and ensure accurate identification.

This study discovers that subcellular localization, protein structural localization, and electrostatic microenvironments dictate cysteine residue reactivity, offering insights into compartment-specific redox regulation in hepatic metabolic disorders. The present redoxome strategy broadens the landscape of the liver cysteine proteome, providing a technique to identify potential therapeutic cysteine targets for liver diseases. Current therapies targeting oxidative stress have failed against various diseases, particularly in cancer (64), nonalcoholic steatohepatitis (65), and type 2 diabetes (66). We believe that understanding cysteine reduction, in addition to cysteine oxidation, may help develop effective therapies for manipulating cysteine redox status, and thereby contribute to drug development against diabetes and its complications.

## Experimental procedures

### Animals

C57BL/6J WT male mice were obtained from Sankyo Lab Service. Mice were fed for 16 weeks with an NCD, CFR-1(Oriental Yeast), or an HFHSD, D03062301 (Research Diet). Mice were housed at 25 °C on a 12-h light/12-h dark cycle with *ad libitum* access to water and food at the Animal Research Facility. All animal care and experiments were reviewed and approved by the ethical committee of Kanazawa University.

### Tissue preparation

Liver samples were collected from 8 independent C57BL/6J WT male mice (4 NCD group, 4 HFHSD group), and each of the 8 liver samples underwent independent proteomic preparation and analysis. After anesthesia, mice were sacrificed for liver tissue isolation of liver. Liver tissue samples were lysed using a chilled-lysis buffer containing catalase (1 mg), EDTA-free protease inhibitor (Roche), and 1× lysis buffer solution (63). Samples were homogenized using a pestle motor on ice until complete protein extraction was ensured. Samples were sonicated at 30 W and 20 kHz during 10 pulses on ice and then centrifuged at 15,000*g* for 30 min at 4 °C. Supernatants were collected, and the protein concentration was determined using a bicinchoninic acid assay.

### Differential alkylation-labeling assay

Unless stated, all steps were performed in the dark, as previously described (53). Protein sample (3 mg) of protein sample was blocked with a thiol alkylation using 24 mM NEM (Fujifilm) in PBS (pH = 7.3), with incubation at 37 °C for 1 h. Protein was precipitated using methanol and chloroform (67) and the resulting pellet, containing the reversibly oxidized cysteine residues, was resuspended in a 5 mM tris(2-carboxyethyl) phosphine (Fujifilm) buffer (pH = 7.8) to remove the reversibly oxidized cysteine residues 30 min at 56 °C, followed by 15 min at room temperature. BPM (Dojindo) (2.5 mM) was added to the previous buffer to label the reversibly oxidized cysteine residues for 1 h at 37 °C. To ensure all cysteine residues were labeled, 5 mM tris(2-carboxyethyl) phosphine buffer and 2.5 mM BPM were added to the previous solution and incubated for 1 h at 37 °C. Labeled proteins were then precipitated with methanol and chloroform.

### Trypsin digestion of labeled proteins

Digestion was performed as described previously (31). Briefly, trypsin digestion buffer containing 50 mM Tris–HCl (pH = 8.0), 4 mM 1,4-DTT (Fujifilm), and 8 M urea was added to the protein pellet. Labeled proteins were sonicated at 30 W and 20 kHz during 10 pulses on ice and then incubated at 60 °C for 1 h. Next, samples were diluted with 50 mM Tris–HCl (pH = 8.0) to decrease the urea concentration to 1 M. The protein concentration was quantified using a bicinchoninic acid assay, and 300 μg of proteins were used together with sequencing-grade trypsin (Promega) in a protease: protein ratio of 1:60 (w/w), and incubated overnight at 37 °C. To maximize the digestion, additional trypsin was added to a final protease: protein ratio of 1:60 (w/w) and incubated for 4 h at 37 °C. Trypsin activity was stopped by adding 0.1% TFA (Sigma-Aldrich) (pH = 1.0) to the digestion mix until the pH decreased to <4. Finally, labeled peptides were centrifuged at 20,000*g* for 20 min at room temperature and supernatants were collected in a new centrifuge to be concentrated in a vacuum centrifuge for 18 h.

### LC-MS analysis

Dried labeled peptides, previously mentioned, were resuspended in 0.1% TFA in ultrapure water, and 100 μg of the sample was fractionated using a high pH reversed-phase peptide fractionation kit (Thermo Fisher Scientific). Briefly, the samples solution was added to a spin column, and centrifuged, then the column was washed with ultrapure water, and the sample was eluted with 5%, 10%, 15%, 20%, and 50% acetonitrile (ACN; Wako Pure Chemical). Collected fractions were dried using a vacuum centrifuge and resuspended in 0.1% formic acid (Wako Pure Chemical). Then, each fraction was column purified (UFC30SV00; Merk Millipore). Purified peptides from samples were loaded and separated on the aurora column (25 cm × 75 μm, 1.7 μm C18; IonOpticks) with a linear ACN gradient (0%–40%) in 0.1% formic acid at a flow rate of 300 nl min^−1^. Peptide ions were detected using a Q Exactive Plus Orbitrap mass spectrometry (MS) (Thermo Fisher Scientific) in the data-dependent acquisition mode with the installed Xcalibur software ver. 4.4 (https://www.thermofisher.com/order/catalog/product/OPTON-30967) (Thermo Fisher Scientific). Full-scan mass spectra were acquired in the MS over 375 to 1500 *m/z* with a resolution of 70,000. The tandem mass spectrometry (MS/MS) searches were conducted using SEQUEST HT search algorithms against the UniProt Mouse protein database using Proteome Discoverer (Version 3.0; Thermo Fisher Scientific). Five fractions (5%, 10%, 15%, 20%, and 50% ACN elutions) were analyzed as one sample. Protein identification was also performed with PD 2.2 using precursor ions quantifier nodes. The processing workflow included spectrum files RC, spectrum selector, SEQUEST HT search nodes, percolator, ptmRS, and minor feature-detector nodes. Methionine oxidation, cysteine NEM, and cysteine BPM were set as variable modifications, and mass tolerances in MS and MS/MS were set at 10 ppm and 0.6 Da, respectively. Trypsin was specified as the protease, and a maximum of two missed cleavages were allowed. Target-decoy database searches were used to calculate the false discovery rate (FDR), and the peptide identification FDR was set at 1%.

### Label-free quantification of proteins using MS data

Label-free quantification was also performed with PD 3.0 using precursor ions Quantifiler nodes. The consensus workflow included multi-sequence format (MSF) files/Feature Mapper/precursor ion quantifier, and MSF Files/peptide-spectrum match groper/peptide validator, peptide and protein filter, protein scorer, protein marker, protein FDR validator, protein grouping, peptide in protein. Normalization of the abundances was performed using total peptide amount mode. The significant change of protein abundance was set by log2 fold change cut off 1.0 with *p* value < 0.05. The protein abundance data are plotted as volcano plot (Fig. 1*D*) by using R version 4.4.2 with “ggpubr” version 0.6, “ggplot2” version 3.5.1, and “ggrepel” version 0.9.6 packages.

### Cysteine proteome profile

The proteins considered for the cysteine identification analysis were only those with a high protein FDR confidence. Then, cysteine residues identified in each sample were classified according to their status: oxidized (BPM-tagged), reduced (NEM-tagged), and unlabeled, and the number of proteins was quantified per status. Next, all peptide sequences containing cysteine were given the same 15 amino acid length, aligning the cysteine in the center of the peptide, also the position of the cysteine residue was identified from their corresponding proteins. For the calculation of the % cysteine redox status in each diet group, the number of oxidized, reduced, and unlabeled cysteine residues was divided from the total number of cysteines identified. All labeled peptides were used to build a Venn diagram based on the cysteine redox status and diet.

### Subcellular localization

All cysteine residue sites identified were matched with subcellular location information from the UniProKB and COMPARTMENTS databases (68, 69). The “COMPARTMENTS” database was delimited by the following criteria: mouse, knowledge channel, and confidence levels of 4 and 5. For quantification, all cysteine residue sites that have multiple subcellular localization were considered. For the calculation of the % cysteine redox status per localization, the number of oxidized and reduced cysteine residues was divided into the total number of cysteine residues identified in the corresponding localization.

### Consistent cysteine residue sites identification

The total number of oxidized and reduced cysteine residues per group was evaluated according to their cysteine position. The cysteine positions detected in two of the two biological replicates and under the same redox category were considered as reproducible cysteine residue sites. For the quantification of the reproducible proteins, if a protein has multiple reproducible cysteine residue sites in the exact category, this was counted only once.

### Enrichment pathway analyses

GO and KEGG enrichment pathway analyses were performed by subjecting the proteins belonging to cysteine residue sites independently to a GO category and KEGG pathway analysis using the ProteINSIDE (70).

### Motif analyses

From the list of the exclusive oxidized and reduced cysteine residues per diet, mentioned in the “KEGG enrichment pathway analyses” section, only the cysteine residue sites that did not show a double redox status (oxidized and reduced) were considered for the motif analysis. Analysis of proximal amino acids was accomplished by extracting peptide sequences with a length of 15 amino acids, centered around each labeled cysteine residue site. The pLogo algorithm (71) was used to visualize the inputted aligned amino acid sequences. For each diet, the foreground contains the exclusive oxidized and reduced cysteine residue sites, and the background used was the protein mouse proteomic dataset. Foreground peptide sequences were not subtracted from the background. The amino acid residues with a PWM score ≥3.75 were considered significantly enriched at the position localized relative to the reduced or oxidized cysteine residue.

The PWM values obtained from the pLogo algorithm were plotted in a heat map using R and “tidyverse” version 2.0.0 and “ggplot2” version 3.4.3 packages. A single heat map was created per exclusive reduced cysteine residue sites found in each diet. The amino acid order used in the heat map was organized based on the properties of the amino acids (72).

### Validation of differential alkylation method

The Cys-PTMs sites that were previously documented in the literature and were also identified in this study were validated by using the individual spectra of the detected peptides in Fig. S2. The citations are as follows: glutathione peroxidase 1 (73, 74), Gclc (45, 75), Gclm (75), Fabp1 (76), Cyp2e1 (77), Slc25a20 (78, 79, 80), and Tst (81, 82).

### Physicochemical property analyses of reactive cysteine residues

The pKa, protein structural localization, disulfide bond participation, and percentage of buried values of reactive cysteine residues were calculated by using the python-based PROPKA3 program (83) using the respective protein structure available in UniProKB database.

### Disulfide bond dihedral angle energy of reactive cysteine residues

The disulfide bond dihedral angle energy values of reactive cysteine residues are calculated by using the web-based server (https://services.mbi.ucla.edu/disulfide/) using the respective protein structure available in UniProKB database.

### Structural analysis

The available protein structures of human GLUL (protein data bank [PDB]: 2OJW), and PFN1 (PDB: 1FIL) in the PDB database were downloaded. The protein structures of mouse GLUL, PFN1, PON1, CTSB, and human PON1 and CTSB were downloaded from AlphaFold (84) (https://alphafold.ebi.ac.uk/). Each structure was visualized with PyMOL (https://www.pymol.org/) (Schrödinger Inc. version 3.1.6.1). The PyTM plugin (85) was used to add a sulfenyl (S-OH) onto Cys^53^ in GLUL and Cys^128^ in PFN1. The CHARMM-GUI “PBEQ solver” option (86) was used to add the disulfide bonds between Cys^42^-Cys^353^ in PON1 and Cys^179^-Cys^211^ between CTSB. The PDB2PQR webtool (87) was used to visualize the electrostatic surfaces in Cys^53^ in GLUL and Cys^128^ in PFN1 and PBEQ solver in Cys^42^-Cys^353^ in PON1 and Cys^179^-Cys^211^ between CTSB. For the sulfenylated cysteines (Cys-SOH), customized forcefields parameters were used to calculate the electrostatic potential surface (Fig. S9). The alignment plugin was used to perform superposition of the structures and the computation of the difference of the RMSD of the atomic positions.

### Immunoprecipitation and Western blot analysis

For FABP1 (Accession ID: P12710) and PON1 (Accession ID: P52430) biotinylation status, the labeled proteins are immunoprecipitated by using Pierce streptavidin magnetic beads (Thermo Fisher Scientific, 8817). Then, we performed Western blotting, as previously reported (24), and immunoblotted by FABP1 antibody (Abcam, AB171739) in 1:1000 dilution and PON1 antibody (Abcam, AB126597) in dilution 1:1000 dilution. Normalization of proteins was assessed by using Ponceau S staining (Thermo Fisher Scientific), as previously reported (88).

### Statistical analysis

All data were analyzed using the GraphPad Prism 10 software (https://www.graphpad.com). The bar graphs of Figure 1, *B*–*C* were shown with mean ± SD. Statistical methods were not used to determine the sample size. Statistical differences between the two groups were assessed using unpaired two-tailed student *t* tests. For the violin plots of Figure 4, *D*–*F*, statistical differences among the four cysteine residue categories were assessed using one-way ANOVA tests followed by Tukey’s *post hoc* test. ∗*p* ≤ 0.05.

## Data availability

The mass spectrometry proteomics data have been deposited to the ProteomeXchange Consortium *via* jPOSTrepo with the dataset identifier PXD064130 (89).

Submission details for accessing the dataset are as follows:

Project accession: PXD064130.

Access URL: https://repository.jpostdb.org/preview/1930891063682d3878c36a7.

Access Key: 3845.

## Supporting information

This article contains supporting information (73, 74, 75, 76, 77, 78, 79, 80, 81, 82, 83).

## Conflict of interest

The authors declare that they have no conflicts of interest with the contents of this article.
