# Supplementary material for: Cysteine redoxome landscape in the liver of male mice fed a high-fat high-sucrose diet
Source: J Biol Chem. 2025 Sep 16;301(10):110730. doi: 10.1016/j.jbc.2025.110730 (PMC12547247; doi:10.1016/j.jbc.2025.110730)
Supplement: Supplementary Figures [file mmc1.pdf]

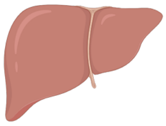

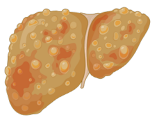

	NCD S1	NCD S2	NCD S3	NCD S4	HFHSD S1	HFHSD S2	HFHSD S3	HFHSD S4
**Peptides (Proteins)**	34,803 (4,707)	19,397 (3,563)	28,118 (4,721)	26,752 (4,568)	49,256 (5,728)	53,568 (5,939)	38,744 (5,011)	40,662 (5,245)
**Cysteine-containing peptides**	1,751	1,720	1,337	1,855	5,190	6,316	3,700	3,914
**Cysteine residue sites**	1,813	1,818	1,385	1,957	5,518	6,791	3,969	4,181
**Oxidized-Cysteines**	161	103	143	165	413	578	318	306
**Reduced-Cysteines**	1,606	1,654	1,170	1,700	4,577	5,570	3,554	3,686

Cysteine residues sites confirmed at least in 2 samples

Redox status	Reproducible cysteines
Oxidized-Cys	115
Reduced-Cys	1363

Redox status	Reproducible cysteines
Oxidized-Cys	377
Reduced-Cys	4026

Supplementary Figure 1. Liver Cysteine redoxome.
The table represent a breakdown of the number of identified peptides, cysteine-containing peptides, cysteine residues sites, oxidized-cysteines, and reduced-cysteines for each sample. From all labeled cysteine residues, those identidied consistently in at least two samples were designated as reproducible cysteines for future comparison between experimental conditions.

A

Accession ID	Protein Name	Peptide Sequence	Cys position	Reference
P11352	Glutathione peroxidase 1	GLVVLGFP[C]NQFGHQENGK	76	(73,74)
P97494	Glutamate-cysteine ligase catalytic subunit	[C]SILNYLK	553	(75,76)
Q3T9P2	Glutamate-cysteine ligase modifier subunit	[C]PSTHSEELR	35	(76)
P12710	Fatty acid-binding protein, liver	NEFTLGEE[C]ELETMTGEK	69	(77)
Q05421	Cytochrome P450 2E1	SLDIN[C]PR	261	(78)
Q7TPW6	Solute carrier family 25 (Mitochondrial carnitine/acylcarnitine translocase)	[C]LLQIQASSENK	136	(79-81)
P52196	Thiosulfate sulfurtransferase	KVDLSQPLIAT[C]R	248	(82,83)

B

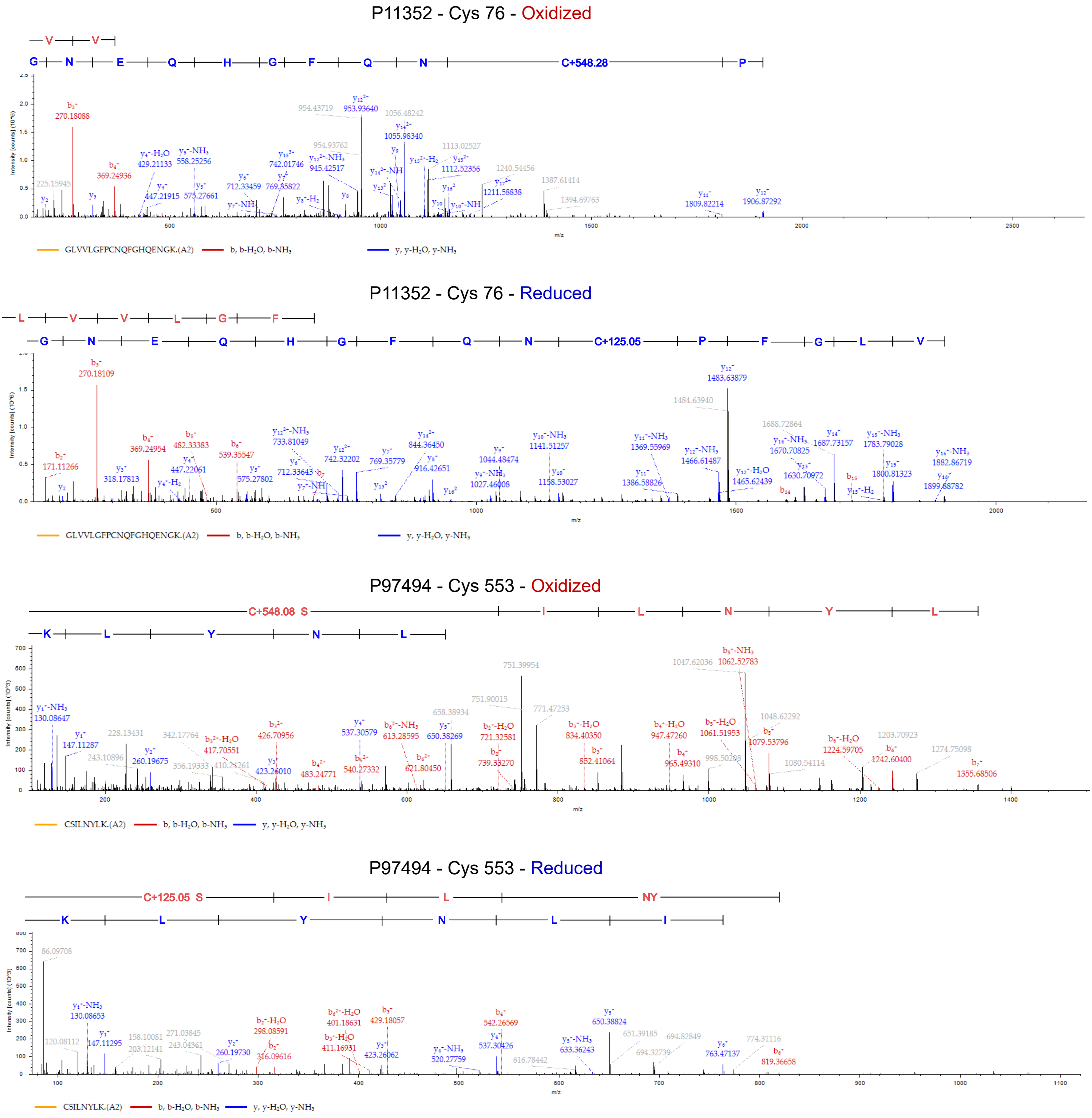

Q3T9P2 - Cys 35 - Oxidized

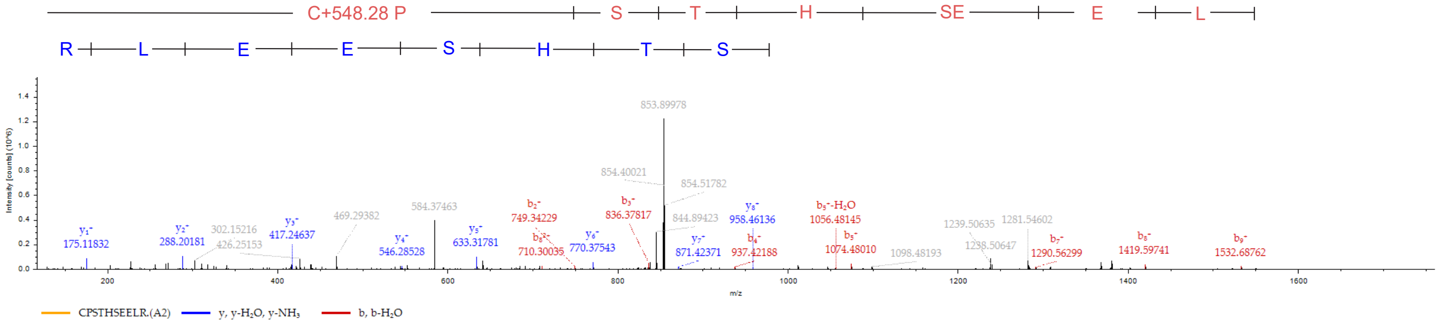

Q3T9P2 - Cys 35 - Reduced

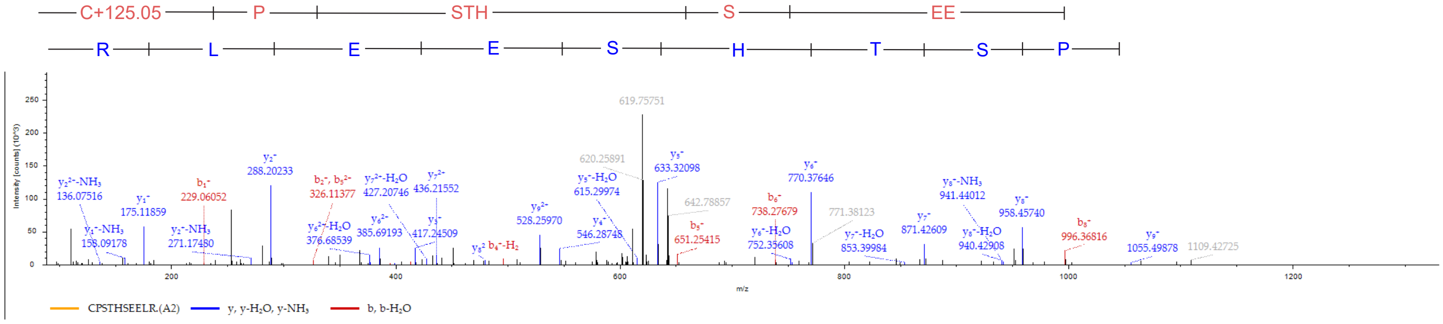

P12710 - Cys 69 - Oxidized

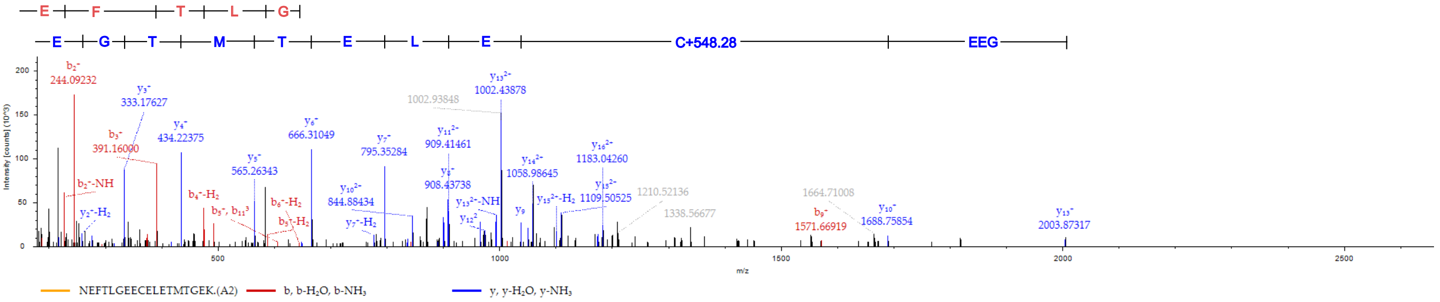

P12710 - Cys 69 - Reduced

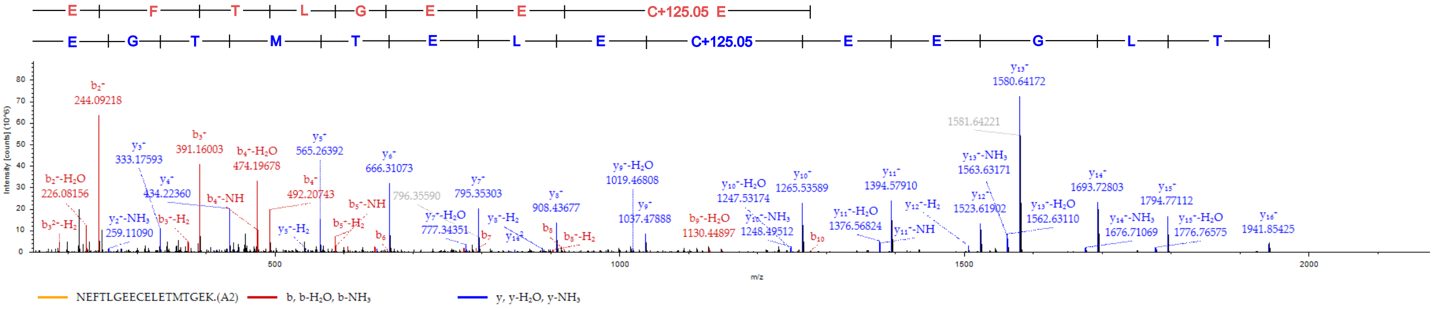

Q05421 - Cys 261 - Oxidized

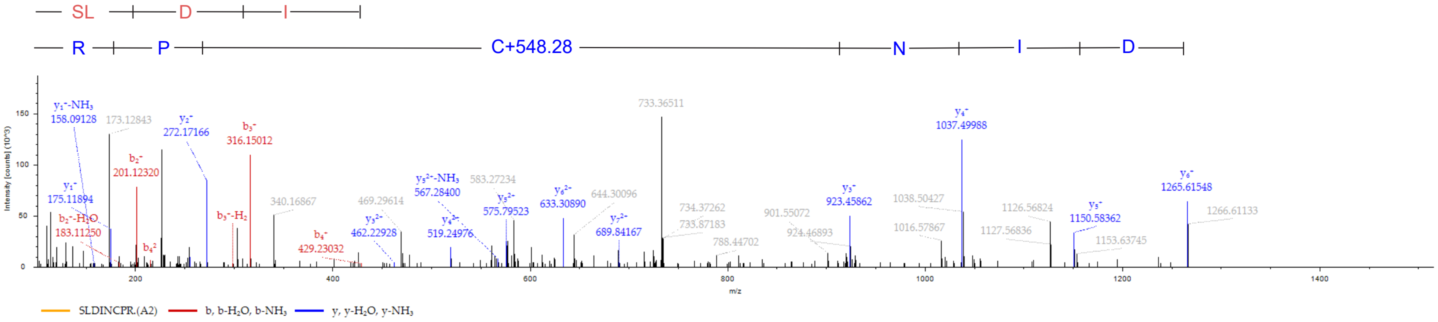

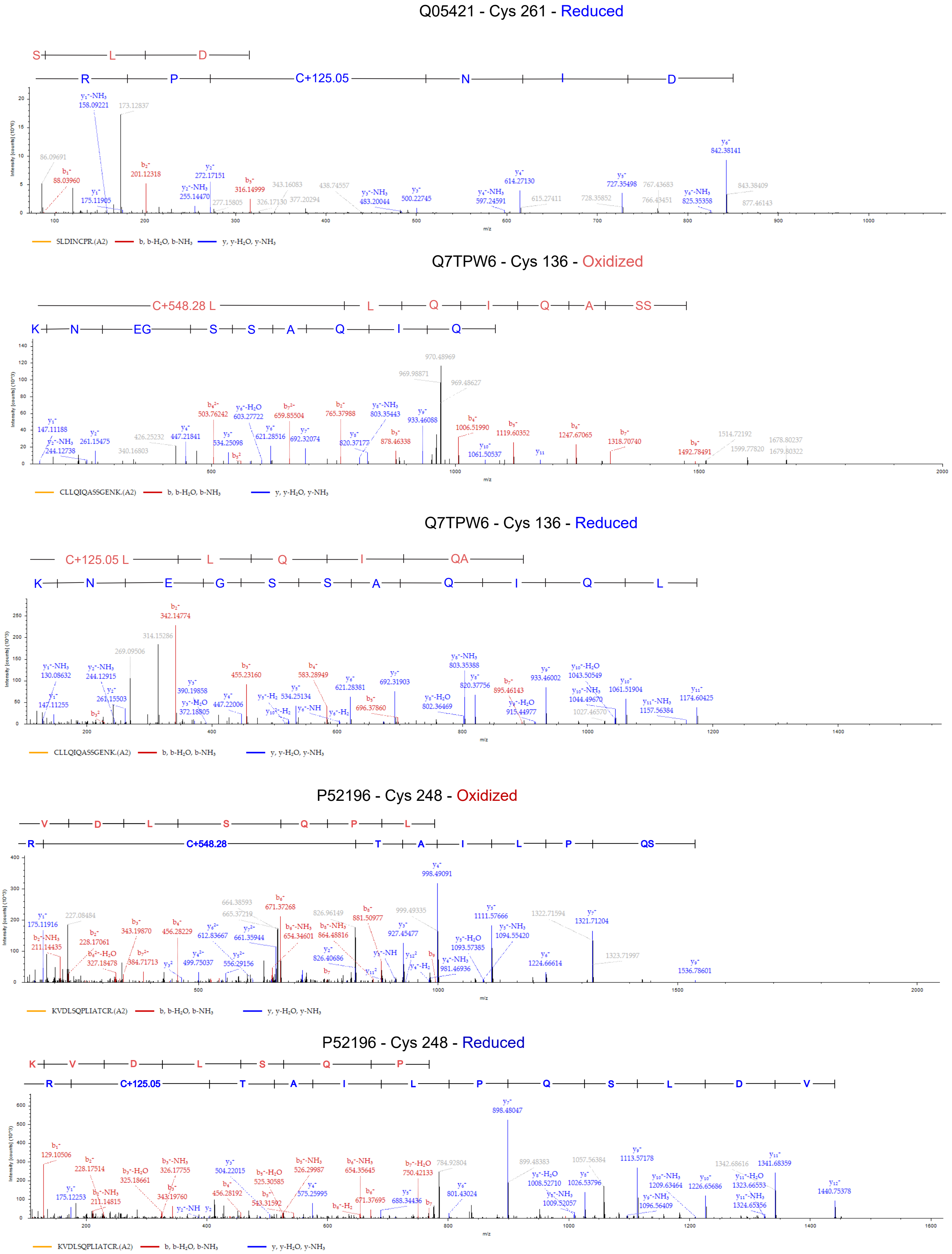

Supplementary Figure 2. Validation of the differential alkylation method.
(A) Table showing Cys-PTMs sites that were previously documented in the literature and were also identified in this study. (B) Individual spectra of the peptides described in (A). C+125 corresponds to a Cys residue alkylated with N-ethylmaleimide (NEM), thus, a Cys that was reduced in cells. C+548.28 corresponds to a Cys residue alkylated with biotin-PECA5-maleimide (BPM), thus, a Cys that was oxidized in cells. The fragment picks of both the b and y ions are shown in the spectra in red and blue respectively. All MS/MS spectra were visualized with Protein Discoverer version 3.0.

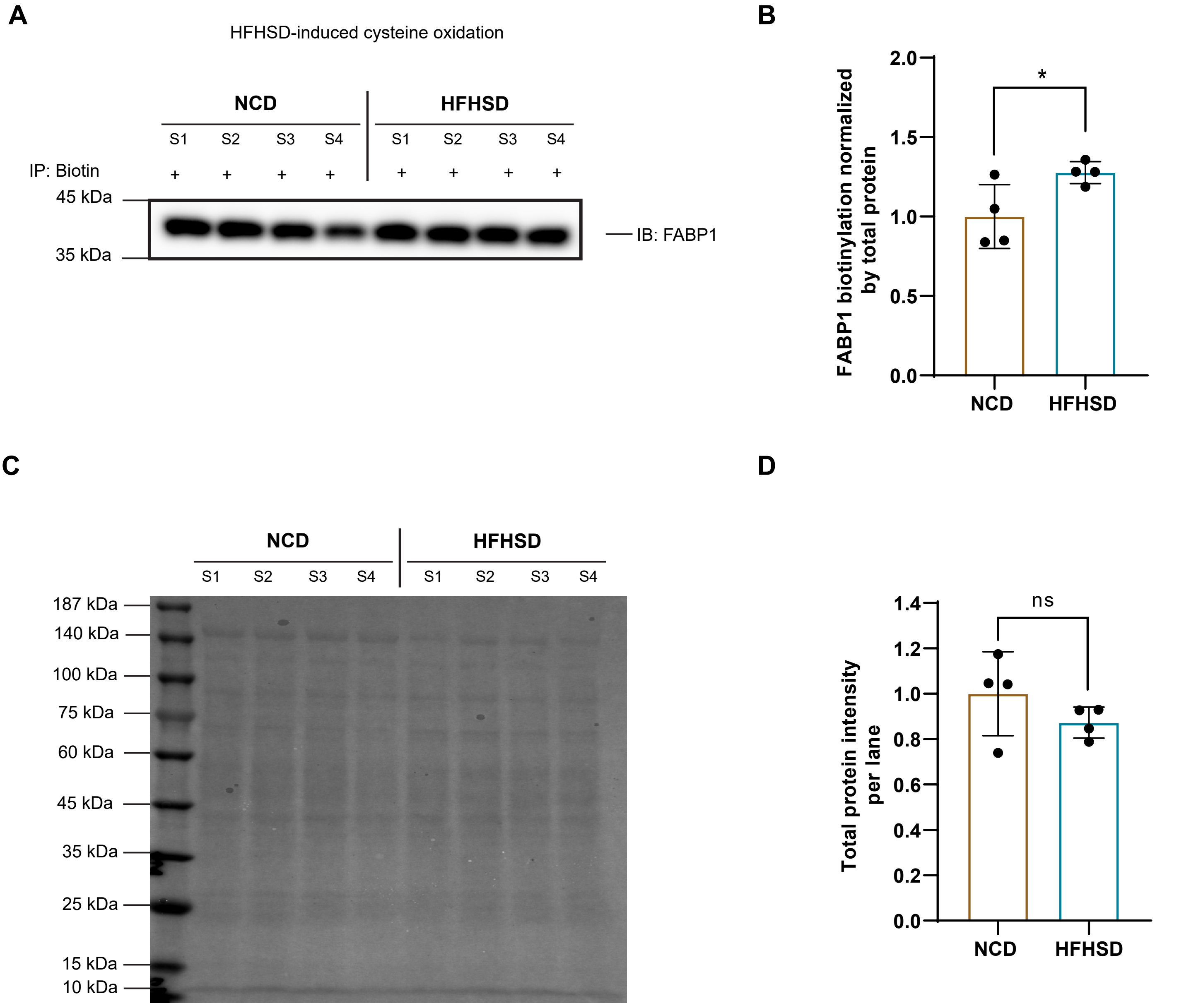

Supplementary Figure 3. Western blot image of HFHSD-induced cysteine oxidation-associated FABP1 biotinylation status. (A and B) Full western blot image (A) and densitometric analysis (B) of HFHSD-induced cysteine oxidation in FABP1 biotinylation status. The liver tissue lysates were labeled with NEM and BPM as described in Fig. 1 step 1 to 55 ug of labeled proteins were used for immuprecipitation by streptavidin, then loaded to SDS-PAGE and immunoblotted by anti-FABP1. (C and D) Full Ponceau S-stained blot images (C) and densitometric analysis per lane (D) of total protein in each sample of (A) after immunoprecipitation. The data in (B) and (D) are shown as mean ± SD. Statistical significance is calculated by two-tailed unpaired student's t-test, *$p \le 0.05$.

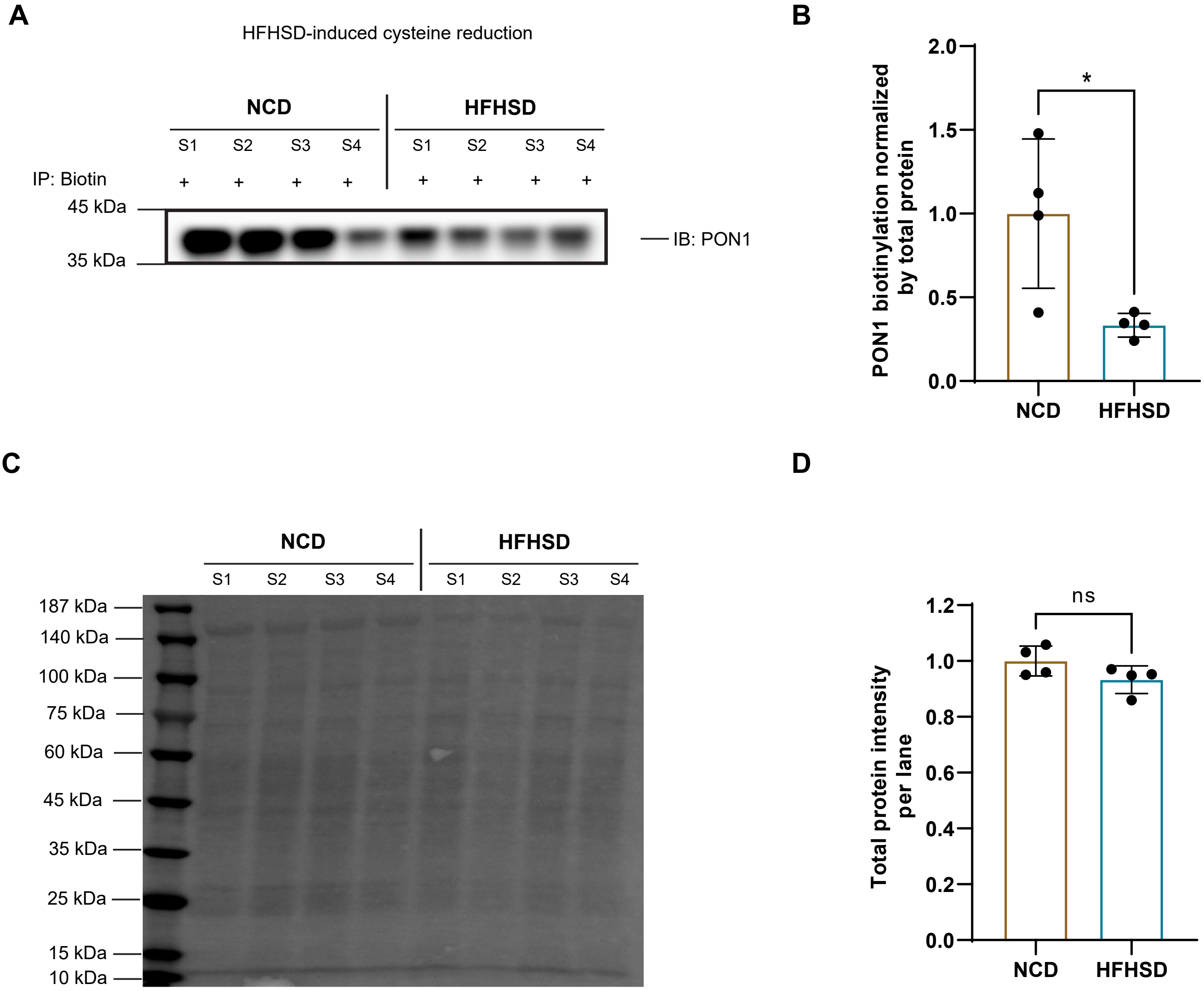

Supplementary Figure 4. Western blot image of HFHSD-induced cysteine reduction-associated PON1 biotinylation status. (A and B) Full western blot image (A) and densitometric analysis (B) of HFHSD-induced cysteine reduction in PON1 biotinylation status. The liver tissue lysates were labeled with NEM and BPM as described in Fig. 1 step 1 to 55 ug of labeled proteins were used for immuprecipitation by streptavidin, then loaded to SDS-PAGE and immunoblotted by anti-PON1. (C and D) Full Ponceau S-stained blot images (C) and densitometric analysis per lane(D) of total protein in each sample of (A) after immunoprecipitation. The data in (B) and (D) are shown as mean ± SD. Statistical significance is calculated by two-tailed unpaired student's t-test, *$p \leq 0.05$.

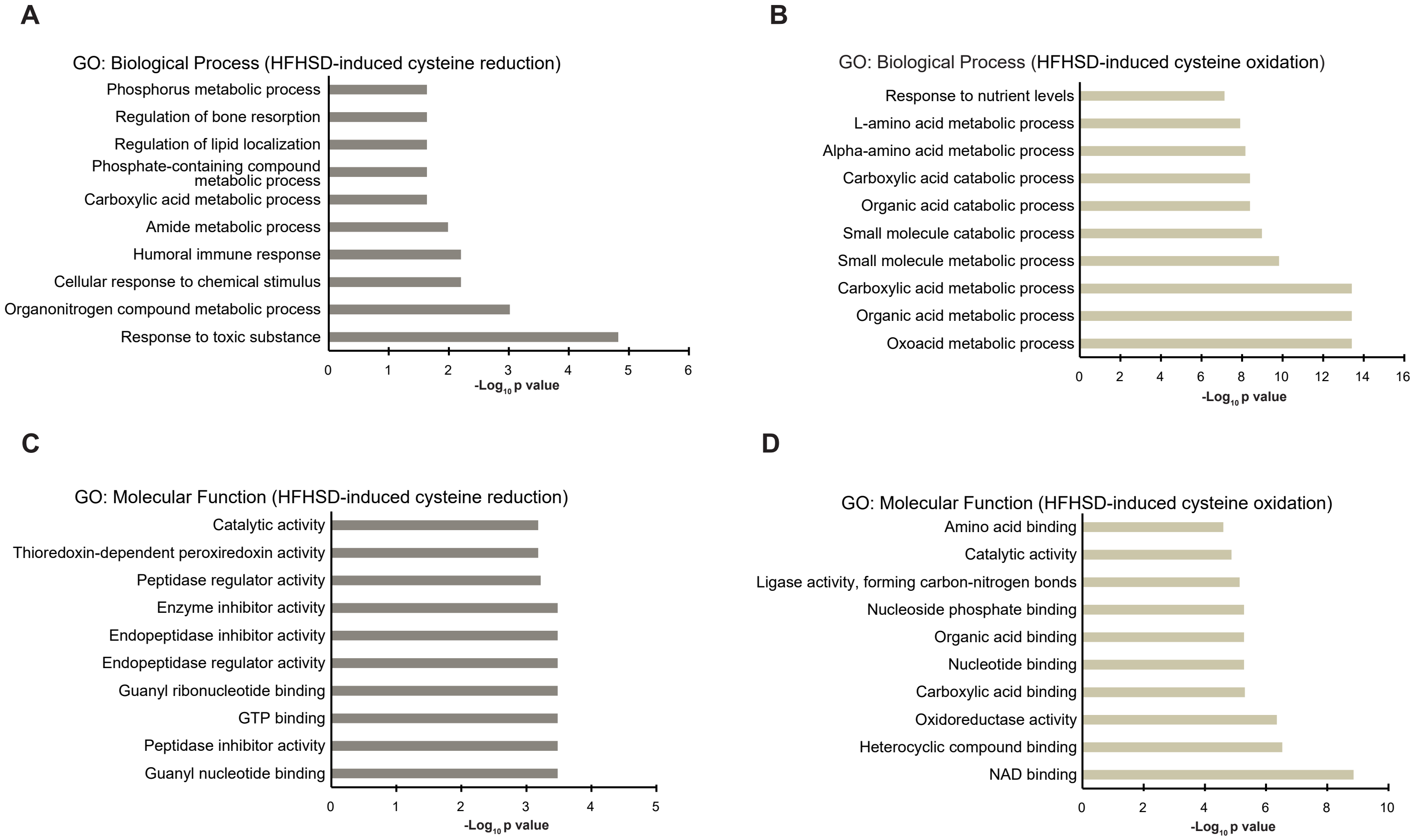

Supplementary Figure 5. Identifying GO terms in HFHSD-sensitive cysteine residues.
(A-D) Enrichment analyseds based on BPs (A-B) and MFs (C-D) found in the HFHSD-induced cysteine reduction and oxidation. The complete lists of entiched terms are presented in Tables S11-S14.

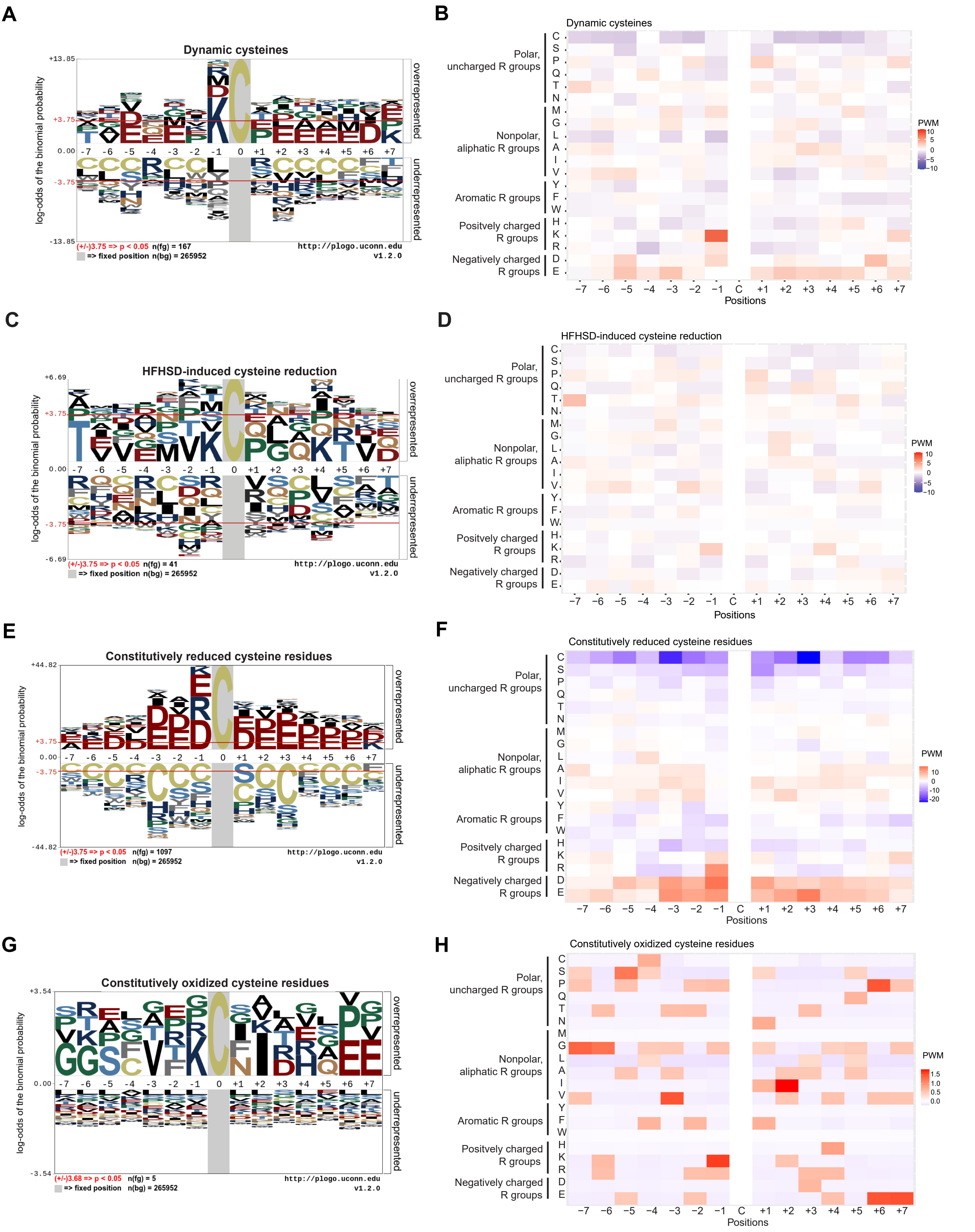

Supplementary Figure 6. Sequence motif analyses of HFHSD sensitive cysteine residues and constitutively oxidized and reduced cysteine residues in NCD and HFHSD. (A) Sequence motif analysis of HFHSD dynamic cysteine residues examining proximal positions (± seven positions) relative to cysteine sites. Horizontal red lines indicate significance at a P-value of 0.05. (B) Heatmap illustrating amino acid sequences HFHSD dynamic cysteine

residues (A). (C) Sequence motif analysis of HFHSD-induced cysteine reduction, examining proximal positions (± seven positions) relative to cysteine sites. Horizontal red lines indicate significance at a P-value of 0.05. (D) Heatmap illustrating amino acid sequences constitutively oxidized cysteine residues in NCD and HFHSD. (E)Sequence motif analysis of constitutively oxidized cysteine residues in NCD and HFHSD, examining proximal positions (± seven positions) relative to cysteine sites. Horizontal red lines indicate significance at a P-value of 0.05. (F) Heatmap illustrating amino acid sequences constitutively oxidized cysteine residues in NCD and HFHSD (E). The heatmap displays position weight matrix (PWM) values, reflecting amino acid frequencies proximal to the cysteine and categorized by amino acid properties. (G) Sequence motif analysis of constitutively reduced cysteine residues in NCD and HFHSD, examining proximal positions (± seven positions) relative to cysteine sites. Horizontal red lines indicate significance at a P-value of 0.05. (H) Heatmap illustrating amino acid sequences constitutively reduced cysteine residues in NCD and HFHSD (G). The heatmap displays position weight matrix (PWM) values, reflecting amino acid frequencies proximal to the cysteine and categorized by amino acid properties.

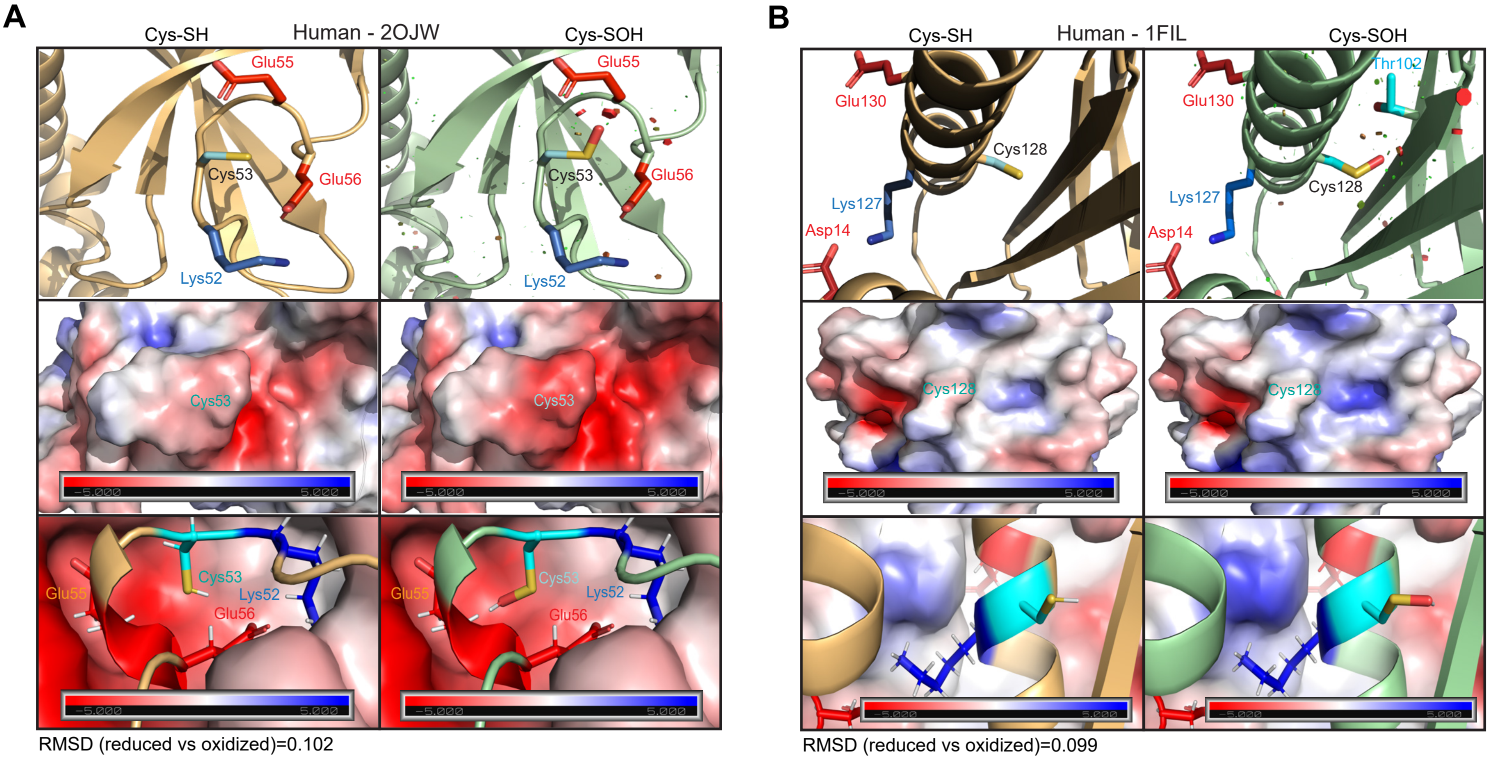

Supplementary Figure 7. Protein structures of HFHSD-induced cysteine oxidation with the KCxE motif. (A) Electrostatic surface potential of GLUL in human (2OWJ). Left: Structure with reduced Cys53 (Cys-SH). Right: Structures with oxidized-Cys53 (Cys-SOH) modeled with PyTMs plugin in PyMOL. The PDB2PQR webtool was used to visualize the electrostatic surface, for oxidized Cys53 (Cys-SOH) a customized AMBER-forcefield parameters were used. Steric van der Waals (vdW) hindrance (vdW clashes) is shown in red. (B) Electrostatic surface potential of PFN1 in human (1FIL). Left: Structure with reduced Cys128 (Cys-SH). Right: Structures with oxidized-Cys128 (Cys-SOH) modeled with PyTMs plugin in PyMOL. The PDB2PQR webtool was used to visualize the electrostatic surface, for oxidized Cys128 (Cys-SOH) a customized AMBER-forcefield parameters were used. Steric van der Waals (vdW) hindrance (vdW clashes) is shown in red. Cys residues are shown in cyan, Lys residues are shown in blue, Glu and Asp residues are shown in red, Thr residues are shown in cyan. Side chains of the amino acids are represented in sticks. RMSD, root mean square deviation of atomic positions.

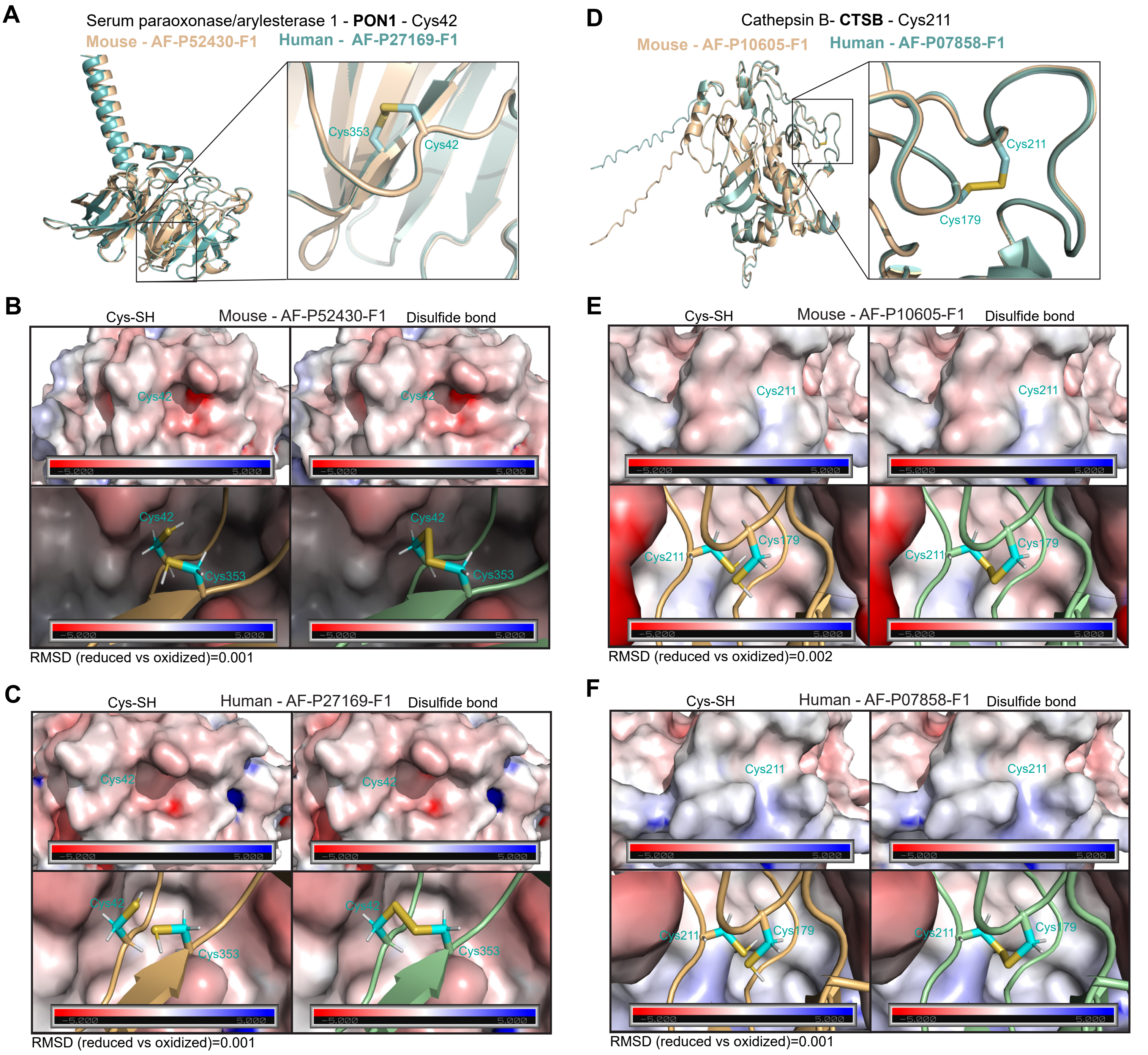

Supplementary Figure 8 . Protein structures of HFHSD-induced cysteine reduction involved in disulfide bond. (A) Overlayed structure of PON1: mouse (Alphafold), and human (Alphafold). Inset shows cysteine residues involved in disulfide bond formation. Disulfide bonds are shown in yellow lines. (B) Electrostatic surface potential of PON1 in mouse (Alphafold). Left: Structure with reduced Cys42 (Cys-SH) modeled with CHARMM-GUI webtool, using PBEQ-solver patches. Right: Structures with oxidized-Cys42(Cys-S-S-Cys) disulfide bond modeled with CHARMM-GUI webtool, using PBEQ-solver patches.The PBEQ-solver webtool was used to visualize the electrostatic surface using default CHARMM topology parameters for cysteine residue and cysteine residue involved in disulfide bond formation. (C) Electrostatic surface potential of PON1 in human (Alphafold). Left: Structure with reduced Cys42 (Cys-SH) modeled with CHARMM-GUI webtool,using PBEQ-solver patches. Right: Structures with oxidized-Cys42(Cys-S-S-Cys) disulfide bond modeled with CHARMM-GUI webtool, using PBEQ-solver patches. The PBEQ-solver webtool was used to visualize the electrostatic surface using default CHARMM topology parameters for cysteine residue and cysteine residue involved in disulfide bond formation. (D) Overlayed structure of CTSB: mouse (Alphafold), and human (Alphafold). Inset shows cysteine residues involved in disulfide bond formation. Disulfide bonds are shown in yellow lines. (E) Electrostatic surface potential of CTSB in mouse (Alphafold). Left: Structure with reduced Cys211 (Cys-SH) modeled with CHARMM-GUI webtool,using PBEQ-solver patches. Right: Structures with oxidized-Cys211(Cys-S-S-Cys) disulfide bond modeled with CHARMM-GUI webtool, using PBEQ-solver patches.The PBEQ-solver webtool was used to visualize the electrostatic surface using default CHARMM topology parameters for cysteine residue and cysteine residue involved in disulfide bond formation. (F) Electrostatic surface potential of CTSB in human (Alphafold). Left: Structure with reduced Cys211 (Cys-SH) modeled with CHARMM-GUI webtool,using PBEQ-solver patches. Right: Structures with oxidized-Cys211(Cys-S-S-Cys) disulfide

bond modeled with CHARMM-GUI webtool, using PBEQ-solver patches.The PBEQ-solver webtool was used to visualize the electrostatic surface using default CHARMM topology parameters for cysteine residue and cysteine residue involved in disulfide bond formation. Cys residues are shown in cyan. Side chains of the amino acids are represented in sticks. RMSD, root mean square deviation of atomic positions.

Forcefield parameters for Cys-SOH to use with PDB2PQR.

```
CSO   N    -0.470000        1.8240     N
CSO   CA   0.070000         1.9080     CT
CSO   CB   -0.110000        1.9080     CT
CSO   SG   -0.050000        2.0000     S
CSO   OD   -0.420000        1.0000     OH1
CSO   H1   0.980000         0.6000     H
CSO   C    0.510000         1.9080     C
CSO   O    -0.510000        1.6612     O
```

Defining name of the CSO (Cys-SOH) residue to use with PDB2PQR

```
<residue>
   <name>CSO</name>
   <atom>
      <name>S</name>
      <useatomname>SG</useatomname>
   </atom>
   <atom>
      <name>OH1</name>
      <useatomname>OD</useatomname>
   </atom>
   <atom>
      <name>H</name>
      <useatomname>H1</useatomname>
   </atom>
 </residue>
```

Supplementary Figure 9. Customized forcefield parameters for Cys-SOH.
The customized forcefield parameters and name definitions are used in AMBER-forcefield to use with PDB-2PQR webtool for the visualization of the electrostatic surface in proteins containing Cys-SOH residue.
